# MicroRNA (miRNA) profiling of maize genotypes with differential response to *Aspergillus flavus* implies zma-miR156–squamosa promoter binding protein (SBP) and zma-miR398/zma-miR394–F -box combinations involved in resistance mechanisms

**DOI:** 10.1007/s44154-024-00158-w

**Published:** 2024-05-10

**Authors:** Prasad Gandham, Kanniah Rajasekaran, Christine Sickler, Harikrishnan Mohan, Matthew Gilbert, Niranjan Baisakh

**Affiliations:** 1 School of Plant, Environmental and Soil Sciences, Louisiana State University Agricultural Center, Baton Rouge, LA 70803 USA; 2 Food and Feed Safety Research Unit, Southern Regional Research Center, USDA-ARS, New Orleans, LA 70726 USA

**Keywords:** *Aspergillus flavus*, Aflatoxin, Coexpression network, microRNA, Resistance, Transcription factor

## Abstract

Maize (*Zea mays*), a major food crop worldwide, is susceptible to infection by the saprophytic fungus *Aspergillus flavus* that can produce the carcinogenic metabolite aflatoxin (AF) especially under climate change induced abiotic stressors that favor mold growth. Several studies have used “-omics” approaches to identify genetic elements with potential roles in AF resistance, but there is a lack of research identifying the involvement of small RNAs such as microRNAs (miRNAs) in maize-*A. flavus* interaction. In this study, we compared the miRNA profiles of three maize lines (resistant TZAR102, moderately resistant MI82, and susceptible Va35) at 8 h, 3 d, and 7 d after *A. flavus* infection to investigate possible regulatory antifungal role of miRNAs. A total of 316 miRNAs (275 known and 41 putative novel) belonging to 115 miRNA families were identified in response to the fungal infection across all three maize lines. Eighty-two unique miRNAs were significantly differentially expressed with 39 miRNAs exhibiting temporal differential regulation irrespective of the maize genotype, which targeted 544 genes (mRNAs) involved in diverse molecular functions. The two most notable biological processes involved in plant immunity, namely cellular responses to oxidative stress (GO:00345990) and reactive oxygen species (GO:0034614) were significantly enriched in the resistant line TZAR102. Coexpression network analysis identified 34 hubs of miRNA-mRNA pairs where nine hubs had a node in the module connected to their target gene with potentially important roles in resistance/susceptible response of maize to *A. flavus.* The miRNA hubs in resistance modules (TZAR102 and MI82) were mostly connected to transcription factors and protein kinases. Specifically, the module of miRNA zma-miR156b-nb – squamosa promoter binding protein (SBP), zma-miR398a-3p – SKIP5, and zma-miR394a-5p – F-box protein 6 combinations in the resistance-associated modules were considered important candidates for future functional studies.

**Supplementary Information:**

The online version contains supplementary material available at 10.1007/s44154-024-00158-w.

## Introduction

Maize (*Zea mays* L.), a major food crop worldwide, is susceptible to *Aspergillus flavus* infection followed by aflatoxin production, especially when the crop is stressed by environmental (abiotic) factors that favor mold growth (Fountain et al. 2014). Colonization of maize kernels by *A. flavus* diminishes grain quality and results in the accumulation of a potentially carcinogenic aflatoxin (Antiga et al. 2020). Aflatoxin, discovered in 1960 following a catastrophic outbreak of turkey “X” disease in the United Kingdom that killed over 100,000 turkey birds, causes a broad spectrum of health hazards in humans and livestock ranging from hepatic failure to central nervous system infections (Hedayati et al. 2007). Aflatoxin related liver cancer deaths are estimated to exceed 100,000 per year, particularly in low- and middle-income countries where corn and peanuts are staple foods (Bandyopadhyay et al. 2016). Factors such as sampling and testing, destruction and disposal, and human and animal health effects can account for much higher total costs associated with aflatoxin contamination.

Aflatoxin contamination of maize grown in the United States resulted in an estimated economic losses of $686.6 million in 2013 (Mitchell et al. 2016). Apart from the cost of testing, the economic loss caused by aflatoxin in 16 U.S. states averaged 17.5 to 24.5 million dollars from 2001 to 2016 (Yu et al. 2020). Under the changing climate scenario, aflatoxin contamination is predicted to cost the U.S. maize industry $52.1 million to $1.68 billion per year (Mitchell et al. 2016). *Aspergillus flavus* can invade maize at any developmental stage, from pre-harvest to storage. Pre-harvest aflatoxin prevention has several advantages including increased crop yield, reduced post-harvest costs, and improved food safety, and this can be achieved by enhancing the maize defense against the fungal pathogen. A few resistant varieties have been developed in the U.S. through conventional breeding. A moderately resistant inbred variety, MI82, was developed from an Indian hybrid commercial cultivar, which showed 74.1% heritability for resistance to aflatoxin buildup in the grain (Maupin et al. 2003). Subsequently, the U.S. Department of Agriculture, Agricultural Research Service, in collaboration with the International Institute of Tropical Agriculture in Nigeria, crossed MI82 with a tropical inbred line 1368 to develop the aflatoxin resistant variety TZAR102 (Menkir et al. 2008).

Based on the kernel screening assay (KSA) with an *A. flavus* GUS transformant to the resistant maize inbred lines MI82 and T115, Brown et al. (1995) have demonstrated that resistance mechanism is independent of the kernel pericarp. A recent metabolome profiling study in response to *A.flavus* infection conducted in maize resistant lines TZAR102, MI82 and susceptible line SC212 revealed significant higher levels of polyamines (PAs) in resistant lines and increased concentrations of glutamate (Glu), glutamine (Gln) and γ-aminobutyric acid (GABA) were observed in susceptible line (Majumdar et al. 2017).

Understanding the underlying genetic and molecular mechanisms involved in maize-*A. flavus* interaction is key to developing maize varieties resistant to the fungus. Several studies investigated the genetic basis of resistance to *A. flavus* infection in maize leading to the identification of a number of QTLs and markers associated with aflatoxin resistance using biparental and genome-wide association mapping (Baisakh et al. 2023). Similarly, several gene expression studies have been conducted to identify differentially expressed genes with potential roles in maize resistance response to *A. flavus*. A recent study using aflatoxin contamination (semi)resistant and susceptible maize lines inoculated with *A. flavus* found strong correlation of flavonoid biosynthesis pathway genes with increased resistance in maize kernels (Castano-Duque et al. 2021). However, results from multiple genomic and transcriptomic studies obfuscate true and consensus marker/gene-trait associations, although identification and validation of consensus genomic regions and genes have been reported in maize via meta-analysis of QTLs, marker-trait associations, and differentially expressed genes (Xiang et al. 2010; Mideros et al. 2014; Baisakh et al. 2023). A meta-analysis of reported QTLs and genes expressed in response to *A. flavus* infection led to pinpointing candidate genes that may control the fungal resistance and aflatoxin contamination (Baisakh et al. 2023). Despite progress in the development of genomics resources and identification of potential molecular mechanisms and factors underlying *A. flavus* resistance in maize, gene regulation of *A. flavus* resistance at the (post)transcriptional level has remained elusive.

MicroRNAs (miRNAs) are a class of non-coding RNAs that regulate gene expression by binding to target mRNAs. miRNAs regulate gene expression by base pairing with complementary sequences on the mRNA molecule, which is guided by RNA-induced silencing complex (RISC) (Nakanishi 2016). The RISC complex then either cleaves the mRNA or inhibits its translation, depending on the degree of sequence complementarity between the miRNA and the mRNA (Ying et al. 2008). Plant miRNAs (19–24 nt) are involved in diverse biological processes including growth and development, signal transduction, and stress responses (Li et al. 2020; Tang et al. 2022). Several plant miRNAs have been shown to play critical regulatory functions in plant defense against fungal infections (Chen et al. 2012; Yang et al. 2013; Mueth et al. 2015; Salvador-Guirao et al 2018a, b). Ongoing research reiterates that miRNAs play crucial roles in plant immunity against pathogens by targeting genes encoding pathogen-associated molecular pattern (PAMP) receptors, transcription factors, defense-related enzymes, and other components of the defense signaling pathway (Islam et al. 2018). In maize, miR393 was the first miRNA reported to be associated with disease resistance via pattern-triggered immunity (PTI) by blocking auxin signaling (Navarro et al. 2006). Differential expression of Zma-miR811, Zma-miR829, Zma-miR845, and Zma-miR408 was observed in response to the pathogenic fungus *Exserohilum turcicum* and Zma-miR811 and Zma-miR829 provided a high degree of resistance to *E. turcicum* (Wu et al. 2014). A recent study reported that Zma-miR408 can enhance disease resistance against *Fusarium verticillioides* ear rot, possibly by regulating the expression of genes involved in plant defense responses (Zhou et al. 2020). These findings highlight the potential role of miRNAs as candidates for improving disease resistance in maize.

Reports on the involvement of miRNAs in resistance against *A. flavus* are limited. In peanut, an integrated approach of profiling the small RNA along with the transcriptome identified that ahy-miR156 might regulate the accumulation of flavonoids in resistant and susceptible genotypes (Zhao et al. 2020). The authors further suggested that Zma-miR482/2118 family could be involved in resistance response, as the NBS-LRR gene target had the higher expression level in resistant genotype. The only experiment in maize that was conducted on two varieties with contrasting aflatoxin resistance reported 21 differentially expressing miRNAs in the resistant variety Mp719 but not in the susceptible variety Va35, which might have role in the resistance response of maize against *A. flavus* (Harper 2018). However, most of the miRNAs reported in this study were computationally predicted and not classified in miRbase. The current study was undertaken to identify candidate miRNAs with possible implications in maize resistance to *A. flavus* through a comparative genome-wide screen of the response to the fungus in susceptible (Va35), moderately resistant (MI82), and resistant (TZAR102) maize genotypes.

## Materials and methods

### Plant materials and fungal inoculation

**Fig. 1:**
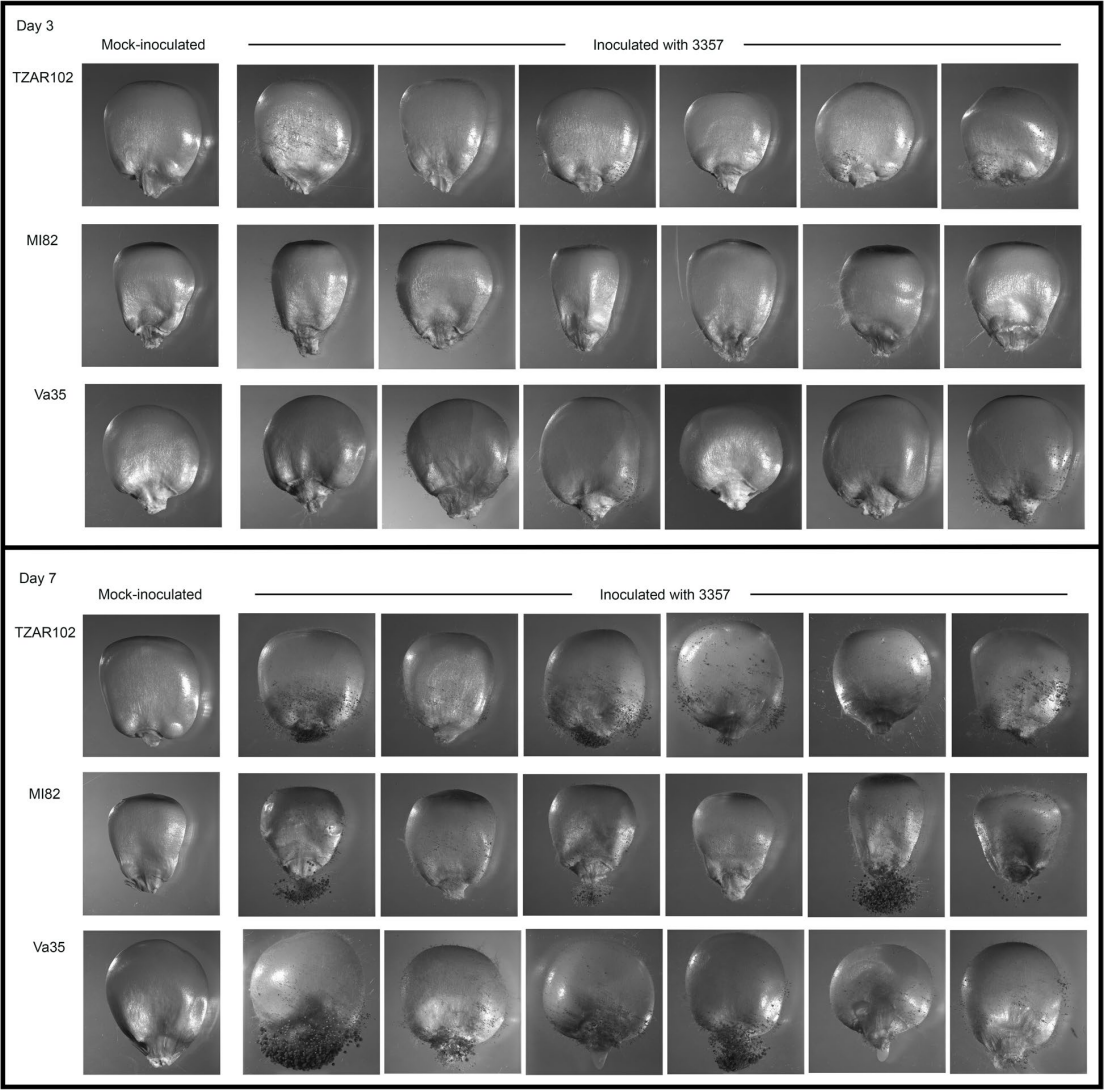
Kernel screening assay (KSA) of maize genotypes TZAR102, MI82 and Va35 showing differential kernel infection response at 3 d and 7 d after inoculation with aflatoxin-producing *Aspergillus flavus* strain NRRL 3357. Mock inoculation was done with 0.02% Triton X-100

Three maize varieties (Va35, MI82, and TZAR102) with different response to *A. flavus* infection (Fig. 1) and/or aflatoxin accumulation were used in the present study. Va35 (USDA NPGS Acc. PI587150) is an inbred line with good agronomic traits, but it shows susceptibility to *A. flavus* and high amounts of aflatoxin accumulation (Kelley et al. 2012). MI82, developed from a hybrid commercial cultivar in India (Brown and Goldman 2016), is a moderately resistant line. TZAR102 (USDA NPGS Acc. PI 654049), which was developed from a cross between a tropical inbred line 1368 and MI82, is resistant to *A. flavus* (Menkir et al. 2008; Brown et al. 2013).

Fungal inoculum was prepared by harvesting conidia in 0.02% Triton X-100 from 6-day-old *A. flavus* toxigenic strain NRRL 3357 (Nierman et al. 2015) grown on V8 medium [5% V8 vegetable juice (Campbell Soup Company, Camden, NJ, United States), 2% agar, pH 5.2] at 31 °C. The maize kernel screening assay (KSA) with the inoculum at 4 × 10^6^ fresh conidia/ml in distilled water was performed in three replications following the method described by Brown et al. (2013) and Rajasekaran et al. (2013). Maize kernels were collected at 8 h, 3 d and 7 d post inoculation to capture the posttranscriptional responses of maize seeds to early fungal infection and fungal growth/establishment and aflatoxin production at a later stage. The kernels were washed with deionized water to remove external mycelia, flash frozen in liquid N_2_ and stored frozen at –80 °C until RNA isolation. Kernels collected after 8 h of mock inoculation with 0.02% Triton X-100 (no fungal spores) served as the control.

### RNA purification and small RNA library preparation and sequencing

Total RNA including small RNA was purified from the kernel samples ground with 0.5 mm diameter zirconia-silica beads (BioSpec Products, Bartlesville, OK) by TissueLyser II (Qiagen, Germantown, MD), using the miRNeasy Mini Kit (Qiagen) as per the manufacturer’s protocol. Following treatment with PureLink DNase (ThermoFisher, Waltham, MA), the RNA was assessed for quality and quantity using Agilent RNA 6000 Nano Kit in a 2100 Bioanalyzer (Agilent, La Jolla, CA).

Altogether, 36 miRNA libraries were made with kernel samples collected from three biological replicates of three genotypes (MI82, TZAR102, and Va35) at four time points (8 h after mock inoculation, and 8 h, 3d and 7 d post inoculation with *A. flavus*). The miRNA library for each sample was prepared from 500 ng of RNA using QIAseq miRNA library kit with miRNA NGS 48 index IL (Qiagen). The library fragment size as determined by 2100 Bioanalyzer with Agilent High Sensitivity DNA Kit (Agilent) was ~ 180 bp. The concentration of the libraries was measured using Qubit dsDNA HS kit on a Qubit fluorometer (ThermoFisher). All 36 indexed libraries were pooled at the final concentration of 1.6 pM and single reads (1 × 75 bp) sequenced at high output mode on an Illumina NextSeq 500 platform (Illumina, San Diego, CA), which generated a total of 365.5 million reads.

### Post-sequencing bioinformatics analyses

**Fig. 2:**
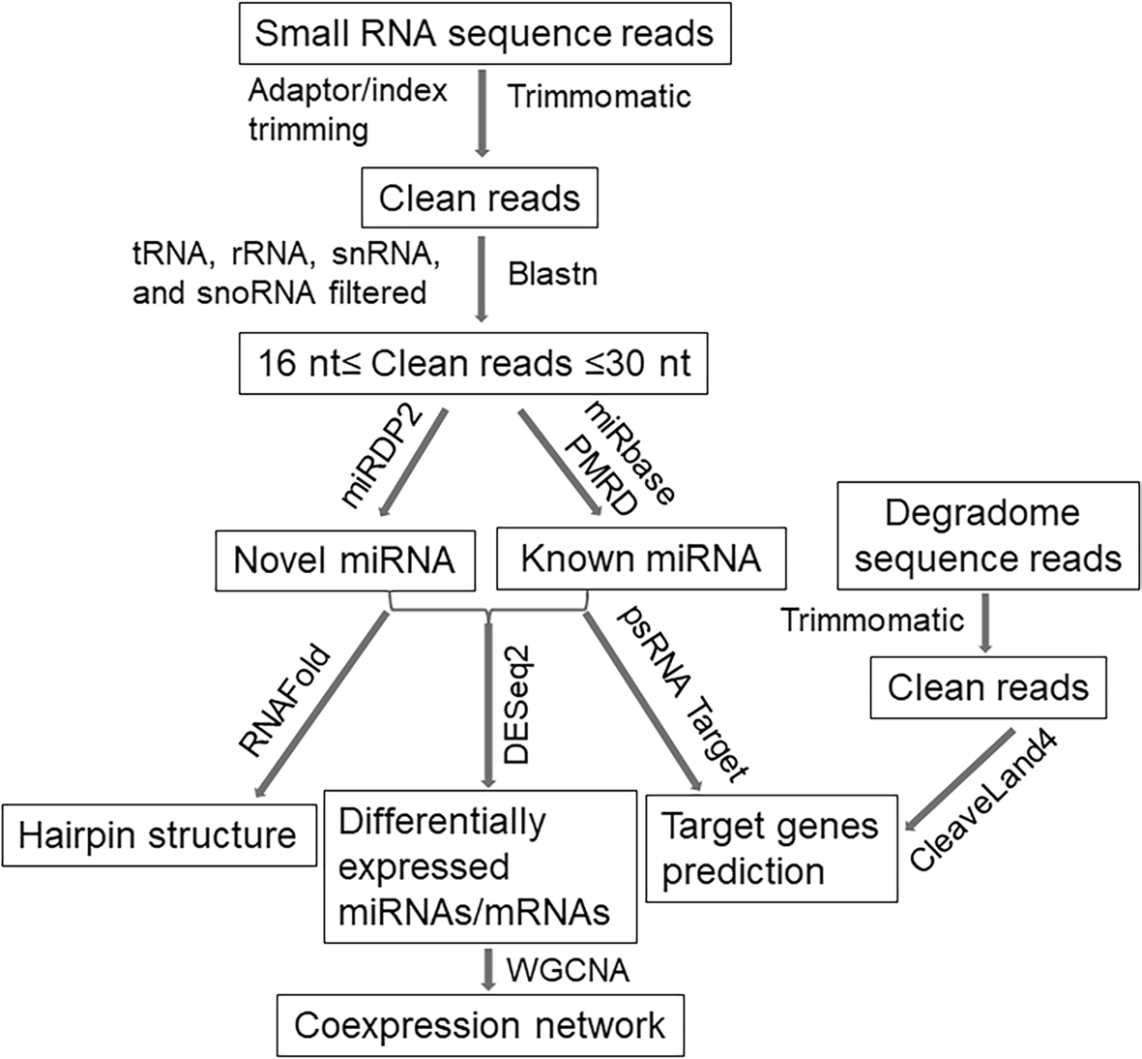
Flowchart of post-sequencing analyses of small RNA reads using various bioinformatics tools

A flowchart showing post-sequencing analyses performed using various bioinformatics tools is shown in Fig. 2. First, raw sequence reads in fastq format were quality checked using FastQC v. 0.11.8 (Andrews 2010). The 5′ and 3′ adapters as well as index sequences were trimmed using cutadapt v3.5 (Martin 2011) and low quality reads were discarded using Trimmomatic v. 0.36 with SLIDINGWINDOW:4:20 (Bolger et al. 2014). Reads with poly A or less than 15 nt were removed. Reads with hits to ncRNA sequences such as lncRNA, snoRNA, snRNA, tasiRNA, tRNA, and rRNA in the Plant Non-coding RNA Database (http://structuralbiology.cau.edu.cn/PNRD/) were also excluded from the downstream analysis (Yi et al. 2015). The filtered, clean reads were blasted against all unique plant miRNAs reference database generated by merging all mature miRNAs from miRbase (Griffiths-Jones et al. 2006) and PMRD (Zhang et al. 2010) by allowing two mismatches to identify known miRNAs in the samples. Unique reads with no hits to known miRNAs were queried to predict putative novel miRNAs using miRDeep-P (Kuang et al. 2019) with Zm-B73-NAM-V5.0 genome (https://www.maizegdb.org/genome/assembly/Zm-B73-REFERENCE-NAM-5.0) as the reference. Stem loop secondary structures of the novel miRNA candidates were predicted from their precursor sequence using Vienna RNAfold (Gruber et al. 2008). The candidate novel miRNAs were named as 'zma-miRX’ followed by a 'number' and ‘-nb’ such as, zma-miRX01-nb, zma-miRX02-nb, etc.

### Identification of differentially expressed miRNAs

To identify the differentially expressed miRNAs (DEMs), read counts from the samples were analyzed by the R package DESeq2 (Love et al. 2014). The miRNAs showing absolute log2-fold change (Log_2_FC) > 1 and < -1 with adjusted p-value (padj) < 0.1 were considered as statistically significant DEMs. The significant DEMs were highlighted in volcano plots generated using EnhancedVolcano R package (Blighe et al. 2023).

Differential expression of miRNAs was determined through semi-quantitative RT-PCR following the protocol described earlier (Zandkarimi et al. 2015). Briefly, first-strand cDNA was synthesized from the purified small RNAs (described under library preparation) using Mir-X miRNA first strand synthesis kit (Takara Bio Inc., San Jose, CA) following the manufacturer’s protocol. RT-PCR was performed using 1 μl of 5x diluted cDNA with the miRNA-specific primers and a universal reverse primer (Supplementary Table S1) of all putative novel miRNAs and DEMs in a total volume of 25 µl. Maize U6 snRNA gene was used as the internal reference.

### Identification of target genes and cleavage sites

The psRNATarget web server (Dai et al. 2018) was used to identify gene targets for the DEMs with a maximum expectation value of 3.5, length for complementarity scoring (HSP size) 19 nt, and two mismatches in seed region. To find the experimentally validated target genes of DEMs, raw degradome sequence data from maize grain, ovary, stalk, tassel, and ear tissues (BioProject accessions PRJNA317519, PRJNA320257, PRJNA733133, and PRJNA208063), publicly available at the NCBI SRA database (https://www.ncbi.nlm.nih.gov/sra), were used. The adapters and low-quality bases from the degradome single-end reads were filtered out using Trimmomatic. The degradome density files were generated by aligning clean reads to the maize reference transcriptome using Bowtie 1.3.1 (Langmead and Salzberg 2012).

Potential cleavage sites on targets of the known and putative novel miRNAs identified in all samples were detected using the publicly available CleaveLand4 pipeline version 4.5 (Addo-Quaye et al. 2009). Based on the comparative abundance of the transcriptome tags relative to the degradome reads matching the target, all targets were divided into 5 categories (0 – 4). Cleavage event hits with p-value ≤ 0.05 and in less than 3 category were considered reliable and used for Target plot (t-plot) generation. The categories 0 to 2 had more than one read aligned at the putative cleavage site on the targets. An interaction network with the known and putative novel miRNAs and their target genes was visualized using Cytoscape version 3.9.1 (Shannon et al. 2003).

### Identification of differentially expressed target genes

Expression of the target genes of DEMs was obtained from the Log_2_FC values reported in the RNA-seq study (Castano-Duque et al. 2021). In addition, the raw RNA-seq sequence reads (SRA Accession No. PRJNA767817) were retrieved from NCBI-SRA database and standard bioinformatics analyses (QC-based filtering and mapping to B73 reference genome) were conducted to obtain FPKM values of the transcripts as described earlier (Bedre et al. 2015).

Differential expression of the target genes was also verified using semiquantitative RT-PCR following the method described by Zandkarimi et al. (2015). Total RNA was isolated from the kernel samples using RNeasy plant minikit (Qiagen) and first-strand cDNA synthesis was performed with iScript cDNA synthesis kit (Bio-Rad, Hercules, CA). SqRT-PCR was performed using primers for target genes (Supplementary Table S2) designed using the primer 3 program and the PCR products were resolved in a 2% 1× TAE-agarose gel. The maize *GAPDH3* gene (glyceraldehyde-3-phosphate dehydrogenase) was used as the internal reference gene.

### Functional enrichment analysis of target genes

The R package topGO (Alexa and Rahnenfuhrer 2021) was used for all target genes of DEMs to identify enriched gene ontology (GO) terms in biological process (BP), molecular function (MF), and cellular component (CC) categories. Also, all target genes were mapped to Kyoto Encyclopedia of Genes and Genomes (KEGG) database to retrieve their annotations that were inputted to clusterProfiler (Yu et al. 2012) to determine the significantly enriched pathways. The GO terms and KEGG pathways with *p* value ≤ 0.05 were considered significantly enriched and depicted as dot plots and horizontal bar plots, respectively.

### Whole genome coexpression analysis

Coexpression network of the DEMs and corresponding differentially expressed target genes was constructed with their Log_2_ (1 + FPKM) values using the WGCNA package of R (Langfelder and Horvath 2008) based on the adjacency matrix deduced from the Pearson’s correlation coefficient (Zhang and Horvath 2005). Hierarchal clustering of genes was performed using the dynamic tree cut algorithm, and network modules (assigned with different colors) were defined after decomposing/combining branches to reach a stable number of clusters (Langfelder and Horvath 2008). The genes (nodes) in an interaction network module were sorted based on the gene weight (Zou et al. 2019) and connected with others according to their connectivity strength. Gene hubs were signified based on higher number of connections at > 0.2 strength and a Kme value higher than 0.8. Cytoscape 3.8.2 (Shannon et al. 2003) and networkD3 v0.1.3 R package with ForceNetwork function were used to have more control for force-directed visualization of complicated network.

## Results

### Overview of small RNA sequencing stats

Sequencing of the 36 libraries prepared from the kernel samples of three maize varieties (TZAR102, MI82, and Va35) at four time points (8 h after mock inoculation and 8 h, 3 d, and 7 d after *A. flavus* inoculation) in three replicates yielded an average of 7.4 million (6.06 to 9.46 million) raw reads with 96.72% reads having Phred quality score ≥ Q30, 50.98% GC content and 76 nt long (Supplementary Table S3). Adapter sequences were trimmed from the reads followed by filtering out the reads with low quality bases, below 18 nt long and with ploy-A tail regions. A total of 42.7% (average 5.37 million reads) of the clean small RNA were 18 to 26 nt long. Length distribution of small RNA reads showed that 24 nt was the most abundant (16.8%) class across all libraries, followed by 22-nt (14.5%) and 21-nt (13.0%) (Supplementary Figure S1). Read length distribution of the small RNA libraries in this study was consistent with several small RNA studies in different tissues of maize that demonstrated predominance of 24 nt class followed by 22 nt small RNA reads (Shen et al. 2013; Liu et al. 2014; Gong et al. 2015; Li et al. 2017; Agapito-Tenfen et al. 2018; Song et al. 2022). Further mapping of the clean reads from 36 libraries showed that 23.33% of the reads aligned to the ncRNAs such as 5S,5.8S, 18S, 23S rRNAs, tRNAs, lncRNAs, snoRNAs, snRNAs, and tasiRNAs (Supplementary Table S3).

### Identification of known miRNAs

Independent BLAST-search of the clean reads unaligned to the ncRNAs from each library against the in-house database built with currently known and experimentally validated 10,948 unique mature miRNAs from 104 plant species from miRbase v21.0 and PMRD identified known miRNAs in each library. Manual examination of miRbase and PMRD database revealed that a few miRNAs have two or more isoforms and miRNAs such as zma-miR444a had different sequences but assigned with the same name. Such isoforms were differentiated by appending an “f” to miR’’ in their sequence ID, such as Zma-miRf444a was given for the duplicate zma-miR444a. Altogether, 275 known miRNAs were identified across the libraries with 241, 233 and 218 known miRNAs from MI82, TZAR102 and Va35 genotypes, respectively. Out of all the known miRNAs, 186 were common among the three genotypes, and 19, 17, and 8 miRNAs were unique to MI82, TZAR102, and Va35, respectively (Supplementary Figure S2).

Of the 275 known miRNAs, 104 (37.8%) matched with known *Zea mays* (zma) miRNAs and the remaining 171 were found to be isoform miRNAs with 100% sequence identity to the miRNAs present in 44 other plant species. As these isoform miRNAs are reported for the first time in maize, they were assigned a name by prefixing with “zma” and suffixing with “-nb”. For example, if a small RNA matched 100% to aau-miR160, it was named as zma-miR160-nb (Supplementary Table S4). Among all the known miRNAs, zma-miR6300-nb had the highest total read counts followed by zma-miR2910-nb, zma-miR166a-3p, zma-miR319b, and zma-miR159a-3p with > 100,000 reads over all samples. More than 50% (164) of known unique miRNAs had greater than 50 reads, which included 130 (53.9%) miRNAs from MI82, 116 (49.7%) from TZAR102, and 122 (55.9%) from Va35.

The 275 known miRNAs belonged to 114 miRNA families where eight families, such as miR166 (26), miR156 (25), miR167 (19), miR159 (15), miR169 (13), and miR171 (11), had more than 10 miRNA members each (Supplementary Table S5). On the other hand, 85 families possessed only one miRNA member. The miRNA families showed markedly different abundances with 33% of 114 miRNA families sequenced more than 1000 times whereas some were detected in less than 10 counts. The diversity of the miRNA families did not have correlation with their abundance. For example, miR166, despite being the most diverse family with 26 members, was fourth in abundance (6.8%) whereas miR6300 with only one member had the highest abundance 21.8%, followed by miR2910 and miR319 with one and five members but 7.7% and 7.4% coverage, respectively (Supplementary Table S6; Supplementary Figure S3). Eighty-three miRNA families had one member each.

### Identification of putative novel miRNAs

Analysis of the clean short reads with no hits to the known miRNA reference database with miRDeep-P identified 41 putative novel miRNA candidates in the maize libraries (Supplementary Table S7). A total of 35, 33, and 38 putative novel miRNAs were identified from MI82, TZAR102, and Va35, respectively of which 31 were conserved across all three genotypes (Supplementary Figure S4). Candidates such as zma-miRX28-nb and zma-miRX36-nb were exclusive to TZAR102, zma-miRX30-nb to MI82, and zma-miRX05-nb, zma-miRX06-nb, zma-miRX21-nb, and zma-miRX24-nb to Va35 (Supplementary Table S7). Twenty-six putative novel miRNAs were very highly represented with more than 1000 counts across all 36 samples, with zma-miRX20-nb, zma-miRX01-nb, zma-miRX25-nb, zma-miRX19-nb and zma-miRX15-nb as the top five in highest to lowest order. The precursor sequence of these newly discovered miRNAs generated thermodynamically stable hairpin structures with a minimum free energy (MFE) ranging from -4.1 to -86.6 kcal/mol (Supplementary Table S7; Supplemental Figure S5).

### Characteristics of the miRNAs

Out of all 316 known (275) and putative novel (41) miRNAs, 163 were 21 nt in length and 42 were 22 nt long (Supplementary Figure S6a). While the known miRNAs ranged from 18 to 24 nt in length, all predicted novel miRNA candidates were 20 nt to 22 nt long. Among the known miRNAs, 21 nt long miRNAs were predominant (150 out of 275) followed by 20 nt (58) and 22 nt (37), whereas 20-nt miRNAs were the most abundant category of novel miRNAs (56.09%; 23 out of 41) followed by 21-nt miRNAs with 13 (31.7%) counts (Supplementary Table S8). The results for the length of the miRNAs are in accordance with a previous miRNA study on incompletely fused carpels in maize (Li et al. 2017). Alignment of the miRNA reads on the Zm-B73-NAM-V5.0 reference genome showed their distribution on all chromosomes and scaffolds, where chromosomes 5 had the highest number of both known (46) and putative novel (11) miRNAs (Supplementary Table S8). The length of the predicted hairpin-like secondary structures of the precursor sequences of novel miRNA candidates varied from 47 to 270 nt, with an average of 148 nt.

The first nucleotide at the 5' end of a miRNA affects its stability and target recognition, and it influences its loading to the effector argonaute (AGO) protein complex for gene silencing through cleavage or translational inhibition (Rogers and Chen 2013). A strong bias towards uracil (U) at the first position of the 5' end of miRNAs has been demonstrated earlier (Mi et al. 2008). One hundred forty-nine (54.2%) of the 275 mature known miRNAs had a first nucleotide bias towards U and 20.4% started with guanine (G) (Supplementary Figure S6b). The nucleotide sequences of the novel miRNA candidates also had U as the most prevalent nucleotide at 43.9% (18 out of total 41) at the 5′ end. The U bias of the first nucleotide at the 5' end was predominant for miRNAs that were 20 nt to 22 nt long (Supplementary Figure S6c).

### Differential expression of miRNAs

**Fig. 3:**
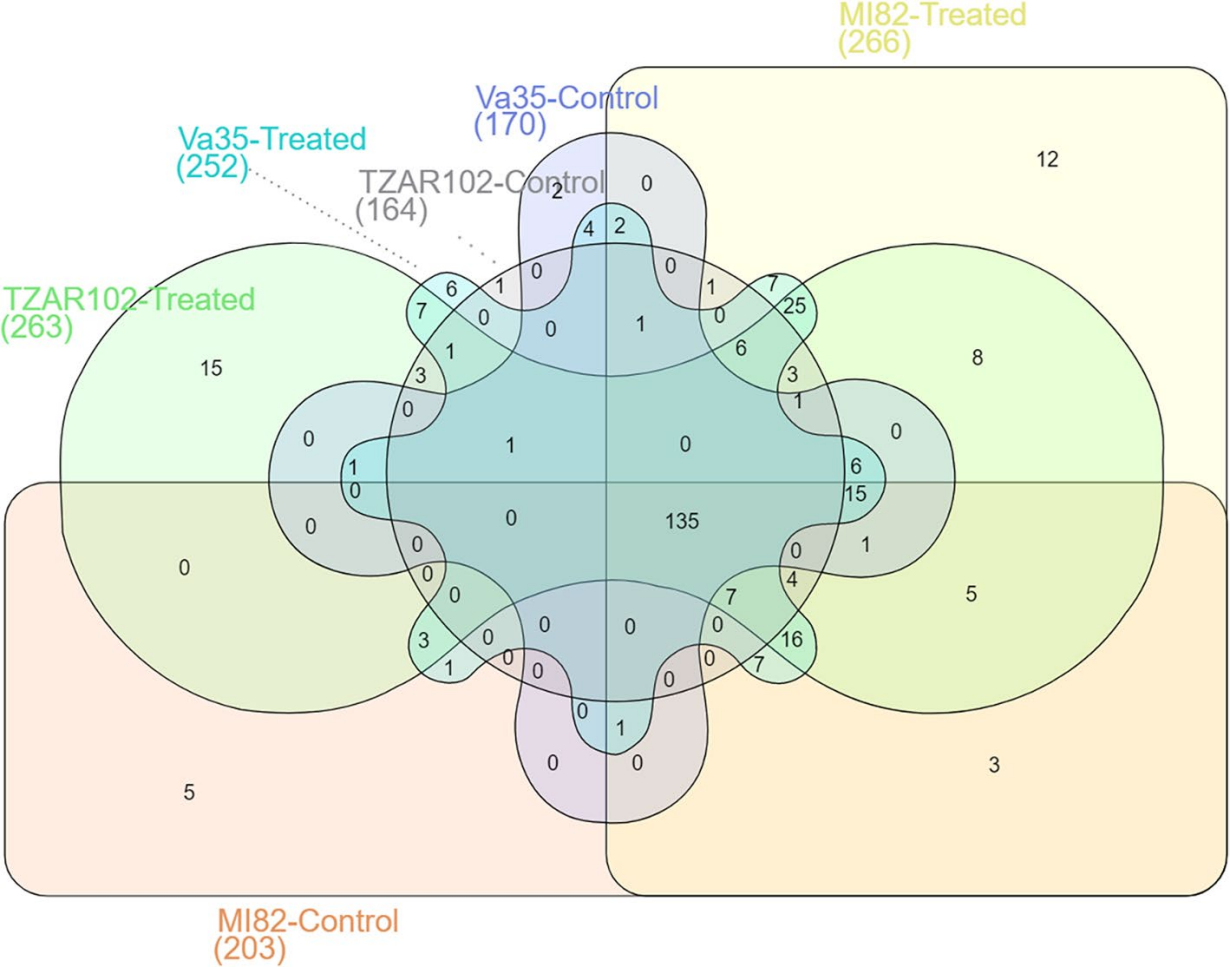
Venn diagram showing miRNAs expressed in three different genotypes (TZAR102, MI82, and Va35) under mock inoculation (control) and *Aspergillus flavus* treatment conditions

Overall distribution of expressed miRNAs shared between and unique to the three genotypes under control (8HMI) and treatment (8HPI, 3DPI, and 7DPI) conditions showed that *Aspergillus flavus* treated maize kernels had a higher number of expressed miRNAs compared to the mock-inoculated control (Fig. 3). A total of 135 miRNAs were found to be commonly expressed between three lines in control and treated kernels. The highest number (266) of expressed miRNAs were observed in the moderately resistant genotype MI82 post-inoculation. Although the lowest number (164) of miRNAs were expressed in mock-inoculated TZAR102, the highest number (15) of miRNAs were specifically expressed in the fungus treated resistant genotype.

**Fig. 4:**
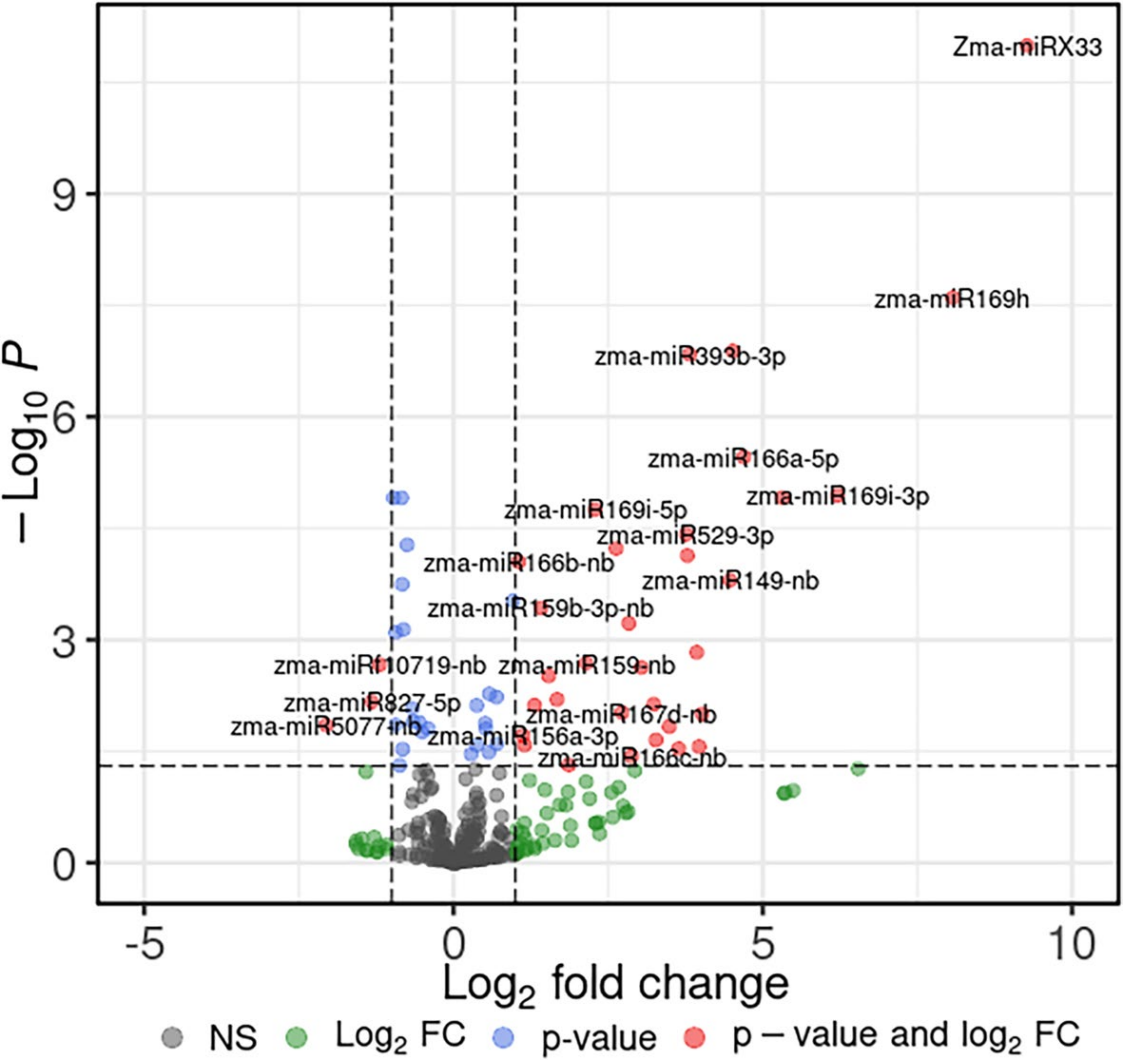
MA plot showing the miRNAs differentially expressed in three different genotypes of maize in response to *Aspergillus flavus* infection. Thirty-nine miRNAs significant up/down regulation at *P* ≤ 0.05 (-Log10*P* ≥ 1.3) are shown as red dots

Differential expressions of all 316 unique miRNAs were evaluated pairwise on normalized read counts across three genotypes at three different time points after *A. flavus* treatment of maize kernels relative to control. A total of 39 (12.3%) miRNAs were significantly differentially expressed (-1 ≤ Log2FC ≥ 1; *Padj* ≤ *0.1*) upon treatment with *A. flavus* irrespective of the genotype and time point (Fig. 4, Supplementary Figure S7). Of these 39 DEMs that belonged to 15 families, 36 were upregulated and three were downregulated. Nine novel candidate miRNAs, zma-miRX31-nb, zma-miRX20-nb, zma-miRX25-nb, zma-miRX03-nb, zma-miRX09-nb, zma-miRX33-nb, zma-miRX11-nb, zma-miRX27-nb, and zma-miRX10-nb were all up regulated by more than 4-fold in treated kernels, of which zma-miRX31-nb was the most upregulated, with 23-fold increase in expression (Supplementary Table S9). The dominant upregulated miRNA families included miR166 with five members, followed by miR169 and miR159 with four members each, and miR156 with three members.

**Fig. 5:**
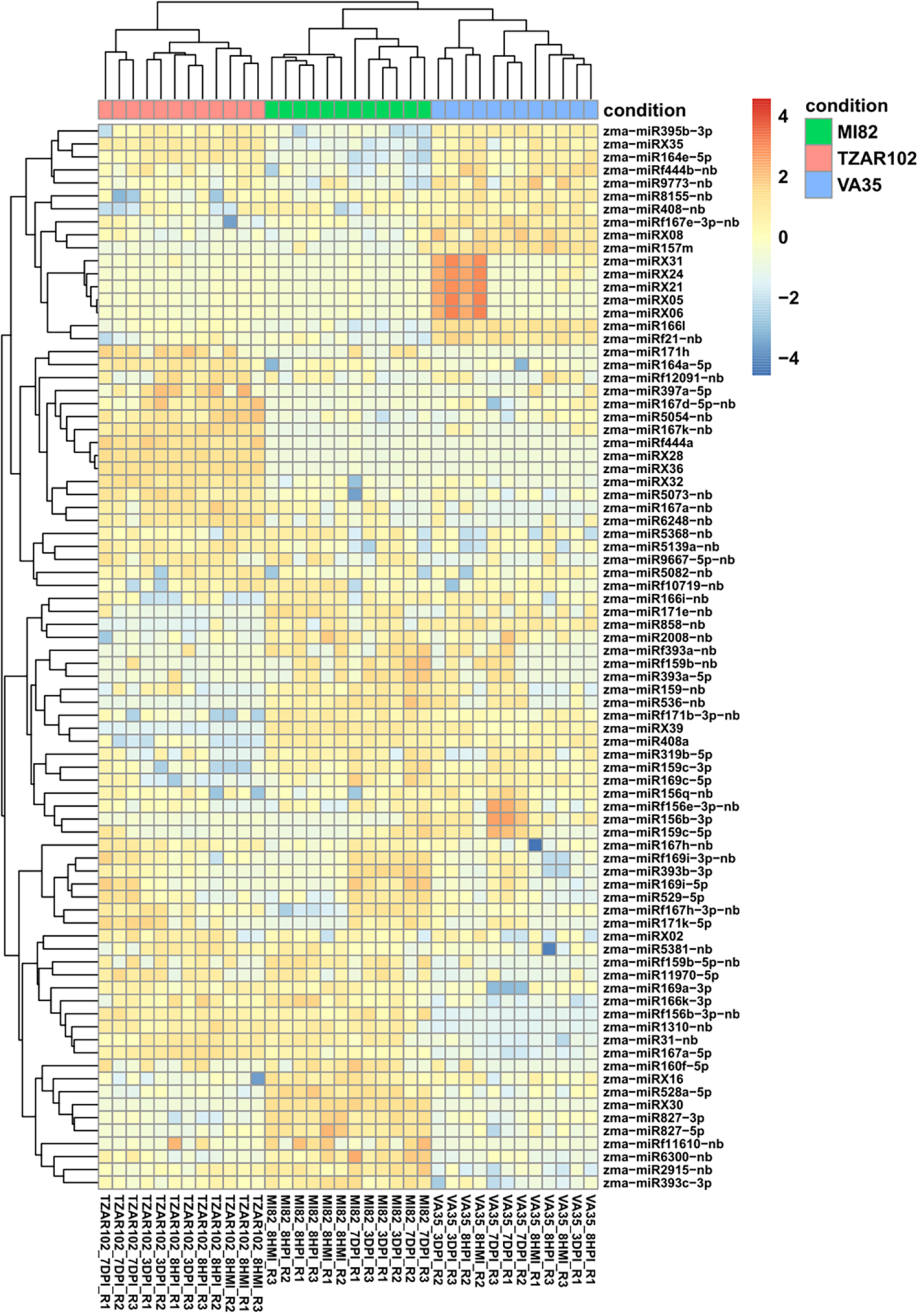
Heatmap showing clustering of three genotypes of maize based on the expression of the miRNAs at different time points in the kernels with or without inoculation with *Aspergillus flavus*

Comparisons between the resistant (TZAR102), moderately resistant (MI82), and susceptible (Va35) genotypes regardless of treatment and time points revealed a total of 82 unique miRNAs significantly differentially expressed, with 49 DEMs (27 up- and 22 downregulated miRNAs) in TZAR102 and 43 DEMs (26 up- and 17 downregulated) in MI82 relative to Va35 (Supplementary Tables S10 and S11). On the other hand, DEMs downregulated (23) were slightly higher than the upregulated ones (19) when TZAR102 was compared against MI82 (Supplementary Table S12). The three genotypes clustered into three distinct clades with TZAR102 samples placed farthest to the Va35 samples (Fig. 5). Three putative novel miRNAs (zma-miRX28-nb, zma-miRX32-nb, and zma-miRX36-nb) and eight known miRNAs (zma-miR5139a-nb, zma-miR167a-nb, zma-miR167k-nb, zma-miR5073-nb, zma-miR6248-nb, zma-miR164a-5p, zma-miR397a-5p, and zma-miRf444a) were upregulated while zma-miRX16-nb, zma-miRX39-nb, zma-miR858-nb, zma-miR536-nb, zma-miR171e-nb, zma-miRf171b-3p-nb, zma-miR156b-3p, zma-miR159c-3p, zma-miR169c-5p, and zma-miR408a were downregulated in the resistant line TZAR102 when compared against the other two lines. At the genotype level, zma-miR395b-3p, zma-miRX35-nb, and zma-miR164e-5p in MI82 and zma-miR166k-3p, zma-miRf156b-3p-nb, zma-miR1310-nb, zma-miR31-nb, and zma-miR167a-5p in Va35 were downregulated.

**Fig. 6:**
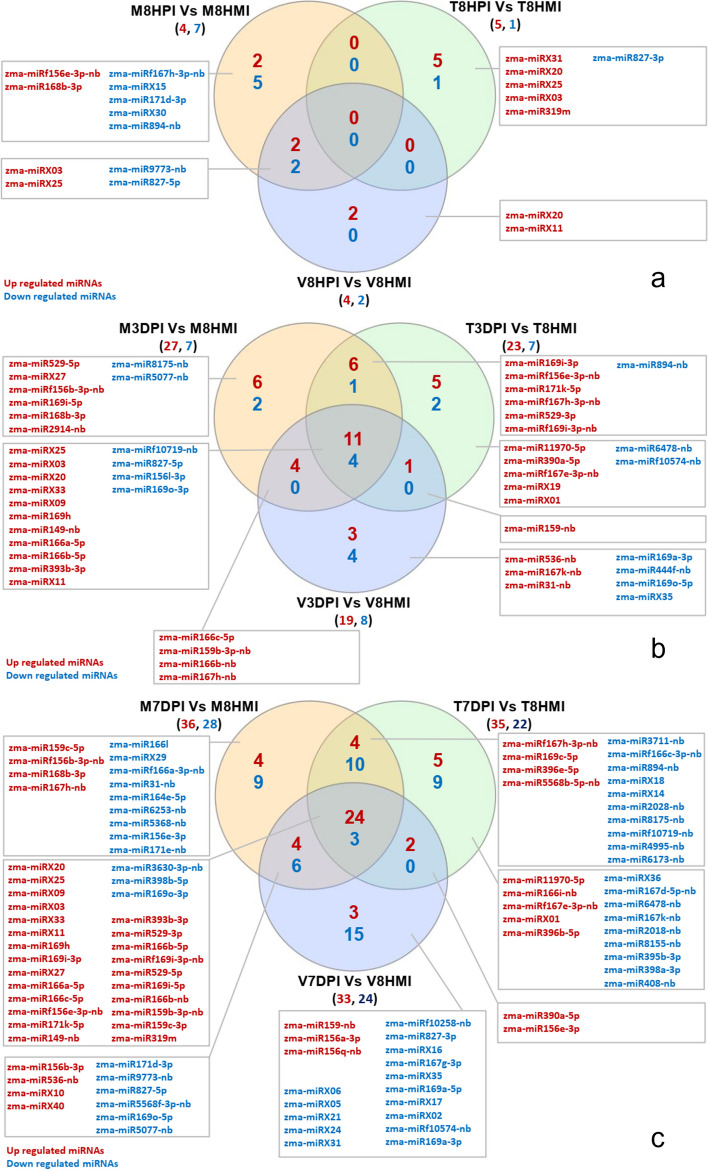
Common and unique miRNAs differentially expressed in three genotypes of maize at 8 h post inoculation (8HPI; **a**, 3 d post inoculation (3DPI; **b**, and 7d post inoculation (7DPI, **c** with *Aspergillus flavus* in comparison to 8 h post mock inoculation (8HMI)

To identify the DEMs at different time points of the three genotypes, the expressions of the miRNAs at 8HPI, 3DPI, and 7DPI were compared against miRNAs expressed at corresponding 8HMI. The results showed that very few DEMs were observed at 8HPI in comparison to control. At 8HPI, zma-miR827-5p and zma-miR9773-nb were downregulated in both MI82 and Va35 (Fig. 6a). At 3DPI, 36 DEMs were upregulated while 13 were downregulated out of which 11 and 4 were commonly up- and downregulated, respectively, across all three lines (Fig. 6b). Most DEMs were observed when comparisons were made for all three genotypes at 7DPI relative to 8HMI, where 64, 57 and 57 miRNAs showed significant differential expression in MI82, TZAR102, and Va35, respectively of which 36, 35 and 33 were upregulated and 28, 22, and 24 were downregulated in that order (Fig. 6c).

The compiled list of miRNAs with the most frequent and significant fold change expression (Table 1) shows that the novel miRNA candidates zma-miRX03-nb and zma-miRX25-nb were consistently upregulated in response to *A. flavus* infection in all three genotypes at all time points. The miRNAs, zma-miRX09-nb, zma-miRX33-nb, zma-miR149-nb, zma-miR169h, and zma-miR166b-5p miRNAs were significantly upregulated in all genotypes between 3 and 7DPI, while zma-miR169o-3p was consistently downregulated. Interestingly, the miRNAs with the highest fold change in expression levels were all putative novel miRNAs such as zma-miRX05-nb, zma-miRX06-nb, zma-miRX21-nb, zma-miRX24-nb, and zma-miRX31-nb with Log2FC values greater than 24.

**Table 1:** Log2-fold change values of differentially expressed miRNAs in three maize genotypes TZAR102, MI82, and Va35 at 8 h, 3 d, and 7 d after inoculation with *Aspergillus flavus*

Genotype	Va35			MI82			TZAR102		
miRNA	8HPI^a^	3DPI	7DPI	8HPI	3DPI	7DPI	8HPI	3DPI	7DPI
zma-miRX03-nb	5.38	8.41	12.4	4.38	11.11	14	3.44	8.39	12.5
zma-miRX25-nb	5.35	8.79	13.84	4	11.4	14.4	4.36	8.49	13.2
zma-miRX09-nb		7.59	12.5		7.36	10.6		6.86	11.5
zma-miRX33-nb		6.96	11.13		8.93	11.1		5.9	9.66
zma-miR149-nb		3.4	4.93		5.87	6.95		4.35	3.86
zma-miR169h		3.66	8.3		6.3	9.05		6.24	10.2
zma-miR166b-5p		3.84	3.92		5.07	5.15		4.11	4.74
zma-miR169o-3p		-4.74	-4.47		-3.64	-2.67		-3.05	-3.38
zma-miR894-nb				-1.1	-1.66	-1.3		-1.39	-1.53
zma-miR159c-3p			1.65			2.32			2.88
zma-miRX31-nb			-35.8				24.72		
zma-miR3630-3p-nb			-1.43			-1.05			-1.9
zma-miR156l-3p		-1.45			-1.81			-2.29	
zma-miR168b-3p				1.37	1.85	1.64			
zma-miR398b-5p			-3.3			-4.5			-3.11
zma-miRX01-nb								1.09	1.41
zma-miRX14-nb						-1.74			-1.29
zma-miRX18-nb						-1.5			-1.49
zma-miRX35-nb		-1.11	-1.62						
zma-miR167k-nb		4.3							-1.39
zma-miR4995-nb						-3.61			-4.96
zma-miR6173-nb						-4.74			-5.07
zma-miRf156b-3p-nb					2.47	2.33			
zma-miRf167e-3p-nb								1.85	2.74
zma-miR3711-nb						-1.18			-1.08
zma-miR6478-nb								-1.07	-1.19
zma-miR5568b-5p-nb						1.24			1.87
zma-miR5568f-3p-nb			-2.28			-4.38			
zma-miR2028-nb						-1.98			-1.23
zma-miR11970-5p								4.62	4.72
zma-miR169a-3p		-1.01	-6.71						
zma-miR169c-5p						1.88			1.7
zma-miR396e-5p						1.78			1.18
zma-miRX05-nb			-29.1						
zma-miRX06-nb			-28.8						
zma-miRX21-nb			-31.4						
zma-miRX24-nb			-32.7						

^a^8HPI 8 h post inoculation with *Aspergillus flavu*s, 3DPI 3 d post inoculation with *A. flavu*s, 7DPI 7 d post inoculation with *A. flavu*s

A three-way comparison of DEMs expressed in TZAR102 vs Va35, TZAR102 vs MI82, and MI82 vs Va35 (mock as well as fungus infected) identified nine miRNAs (zma-miR171h, zma-miR171k-5p, zma-miR167h-nb, zma-miRX02-nb, zma-miR5381-nb, zma-miR408-nb, zma-miR8155-nb, zma-miR159c-5p, and zma-miRf156e-3p-nb) to be specifically differentially expressed in TZAR102 of which the first five were upregulated while the others were downregulated. When compared against Va35, zma-miR171h was significantly highly overexpressed (4.9-fold) in TZAR102 and zma-miR159c-5p and zma-miRf156e-3p-nb were significantly downregulated at 3.19-fold and 3.32-fold, respectively.

### Identification of gene targets for the miRNAs

Five hundred and forty-four genes were predicted as potential targets for 311 out of the 316 unique miRNAs. Seven miRNA families, miR156, miR5568, miR166, miR396, miR167, miR169, and miR171 had the highest number of targets with 220 (38 unique), 41 (35 unique), 205 (30 unique), 40 (28 unique), 39 (28 unique), 51 (23 unique) and 59 (14 unique) targets respectively (Supplementary Table S13.1; Supplementary Figure S8). The 41 putative novel miRNAs predictably targeted 80 genes where zma-miRX19-nb, zma-miRX35-nb, and zma-miRX38-nb topped the list with 8, 6, and 5 possible targets, respectively. A majority of the target genes were members of transcription factor (TF) binding gene families such as APETALA2 (AP2), auxin response factors (ARF), basic leucine zipper (bZIP), basic helix-loop-helix (bHLH), Cys2 His2 zinc finger motifs (C2H2), homeodomain leucine zipper (HD-ZIP), myeloblastosis (MYB), NAM, ATAF1/2, and CUC2 (NAC) transcription factor, nuclear transcription factor Y subunit A-3 (NF-YA), and SQUAMOSA promoter binding protein-like (SBP) of which HD genes such as Zm00001d013699, Zm00001d026325, Zm00001eb000690, Zm00001eb031670, Zm00001eb045630, Zm00001eb050660, Zm00001eb136060, Zm00001eb337970, and Zm00001eb404260 were targeted by 21 different miRNAs (Supplementary Table S13.2). In addition, genes containing SBP domain and GRAS domain were the target for the highest number of miRNAs (Supplementary Table S13.3). The miRNAs post-transcriptionally regulate primarily through mRNA cleavage in plants. Therefore, the cleavage sites of the targets predicted by psRNAtarget were searched using sequences from four different degradome libraires available from different maize tissues. A total of 19,054 (unique 10,636) sliced targets for 273 known and 41 novel miRNAs were identified of which 750 targets were in category I, 752 in category II, and 17,552 targets belonged to category III (Supplementary Table S14; Supplementary Figure S9). Based on the *P* value cut off ≤ 0.05, 256 target genes were identified for 153 unique miRNAs in the degradome libraries.

### Functional enrichment of the target genes

**Fig. 7:**
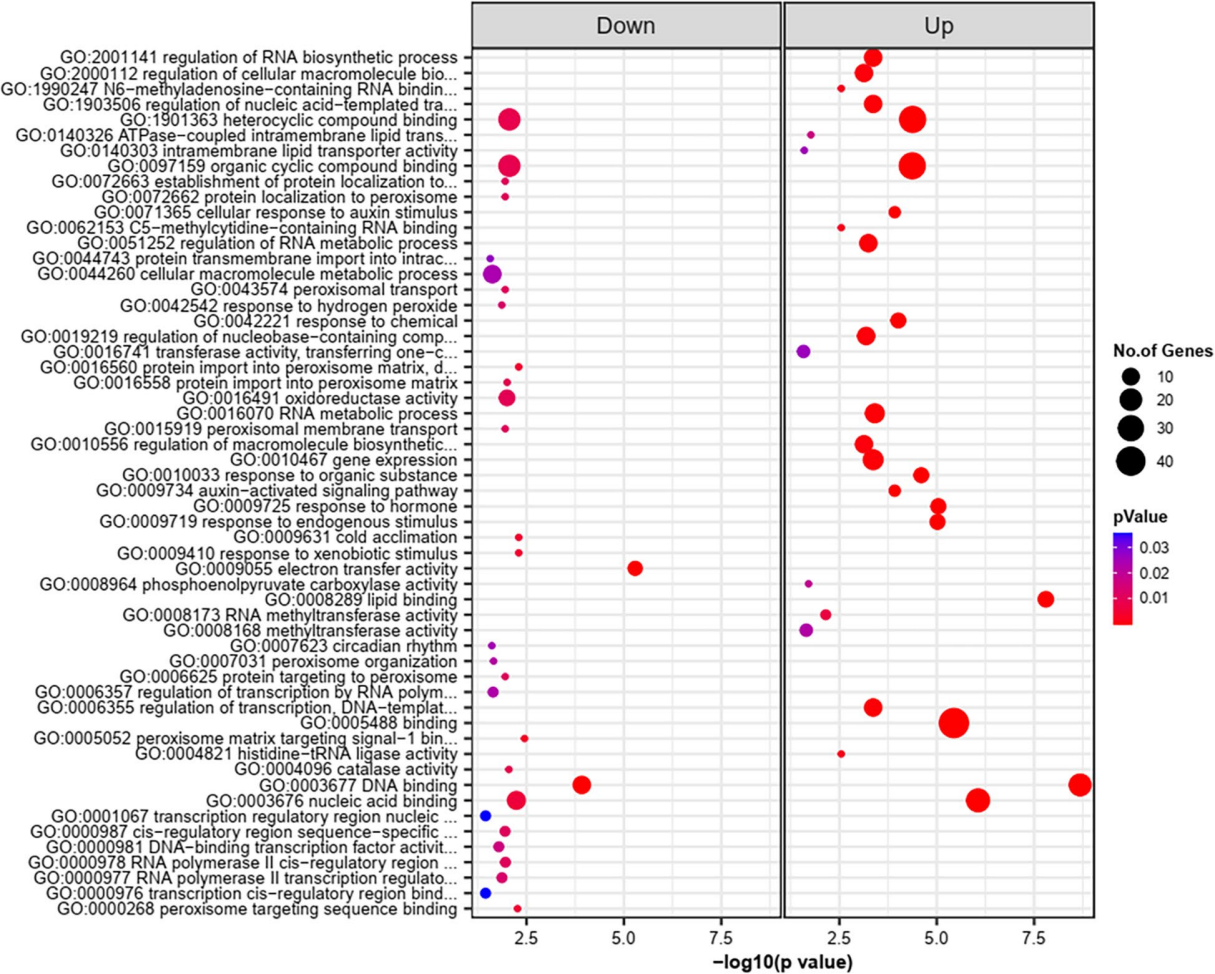
The enriched biological process (BP) and molecular function (MF) GO (Gene ontology) terms annotated to the miRNA target genes in TZAR102 treated vs Va35 treated samples are shown as a dot plot. The color gradient of each dot indicates the P values from Fisher’s exact test, and the size of the dot is proportional to the number of genes associated in the given GO term

Comparative gene ontology (GO) enrichment analysis of the target genes predicted by psRNAtarget and verified in degradome libraries (Supplementary Table S15) showed that GO terms, such as regulation of RNA biosynthetic process (GO:2,001,141), regulation of cellular macromolecule biosynthetic process (GO:2,000,112), regulation of nucleic acid-templated transcription (GO:1,903,506), and heterocycle biosynthetic process (GO:0018130) were suppressed in the biological process (BP) category irrespective of genotype and time point. Two biological processes, peptidyl-threonine phosphorylation (GO:0018107) and cellular protein metabolic process (GO:0044267) were commonly repressed at 3 d post fungal treatment in TZAR102. GO terms in BP such as sulfate assimilation (GO:0000103), lipid translocation (GO:0034204), and phospholipid translocation (GO:0045332) were significantly suppressed in fungus inoculated TZAR102 kernels relative to other two genotypes. In MI82 and Va35 at 3DPI and 7DPI, potassium ion transport (GO:0006813) and potassium ion transmembrane transport (GO:0071805) were the enriched biological processes. On the other hand, two most notable BPs involved in plant immunity such as cellular response to oxidative stress (GO:00345990) and cellular response to reactive oxygen species (GO:0034614) were significantly upregulated in the resistant genotype TZAR102 (Fig. 7).

**Fig. 8:**
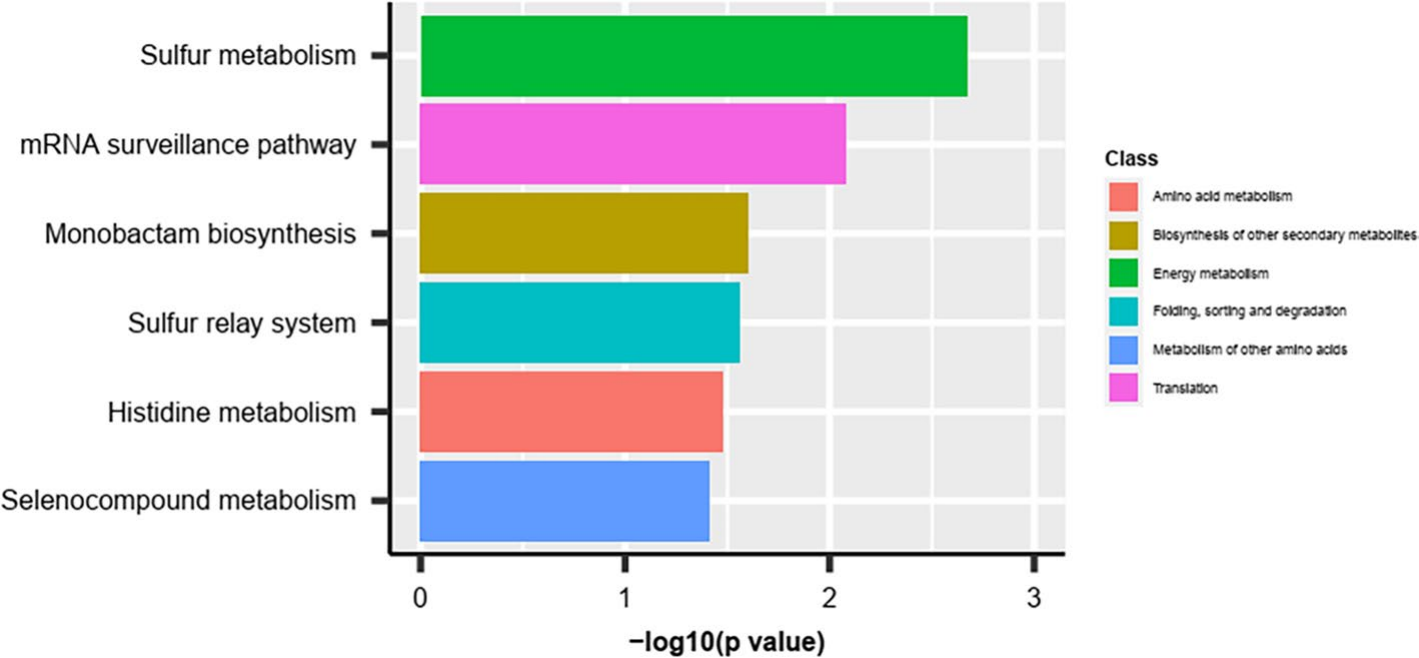
KEGG enrichment analysis of differentially expressed miRNAs (DEMs) of TZAR102 at 7 d post inoculation (T7DPI) vs 8 h mock inoculation (T8HMI)

A Blast2GO database of all *A. flavus* genes revealed that organic cyclic compound binding (GO:0097159) and heterocyclic compound binding (GO:1,901,363) were major binding activities in the molecular function (MF) (Chang 2014). Surprisingly, genes annotated in these two terms were downregulated in all different combinations in our study. In all three genotypes at 3DPI, the soluble N-ethylmaleimide-sensitive factor attachment protein receptors (SNARE) binding (GO:0000149) term was enriched, indicating its significance in the response to the invading pathogen. The cation transmembrane transporter activity (GO:0008324), inorganic cation transmembrane transporter activity (GO:0022890), and sequence-specific double-stranded DNA binding (GO:1,990,837) at 3DPI and protein kinase activity (GO:0004672), protein tyrosine kinase activity (GO:0004713), and kinase activity (GO:0016301) at 7DPI were induced in the susceptible genotype Va35. The most important enriched GO terms in fungus inoculated kernels at cellular component (CC) category were CCAAT-binding factor complex (GO:0016602), intracellular membrane-bounded organelle (GO:0043231), and RNA polymerase II transcription regulation (GO:0090575). Among the genes targeted by DEMs, genes involved in six biosynthetic pathways were significantly enriched, where sulfur metabolism preceded the mRNA surveillance pathway among the top two pathways (Fig. 8).

### Coexpression network and identification of hub genes in miRNA-mRNA modules

**Fig. 9:**
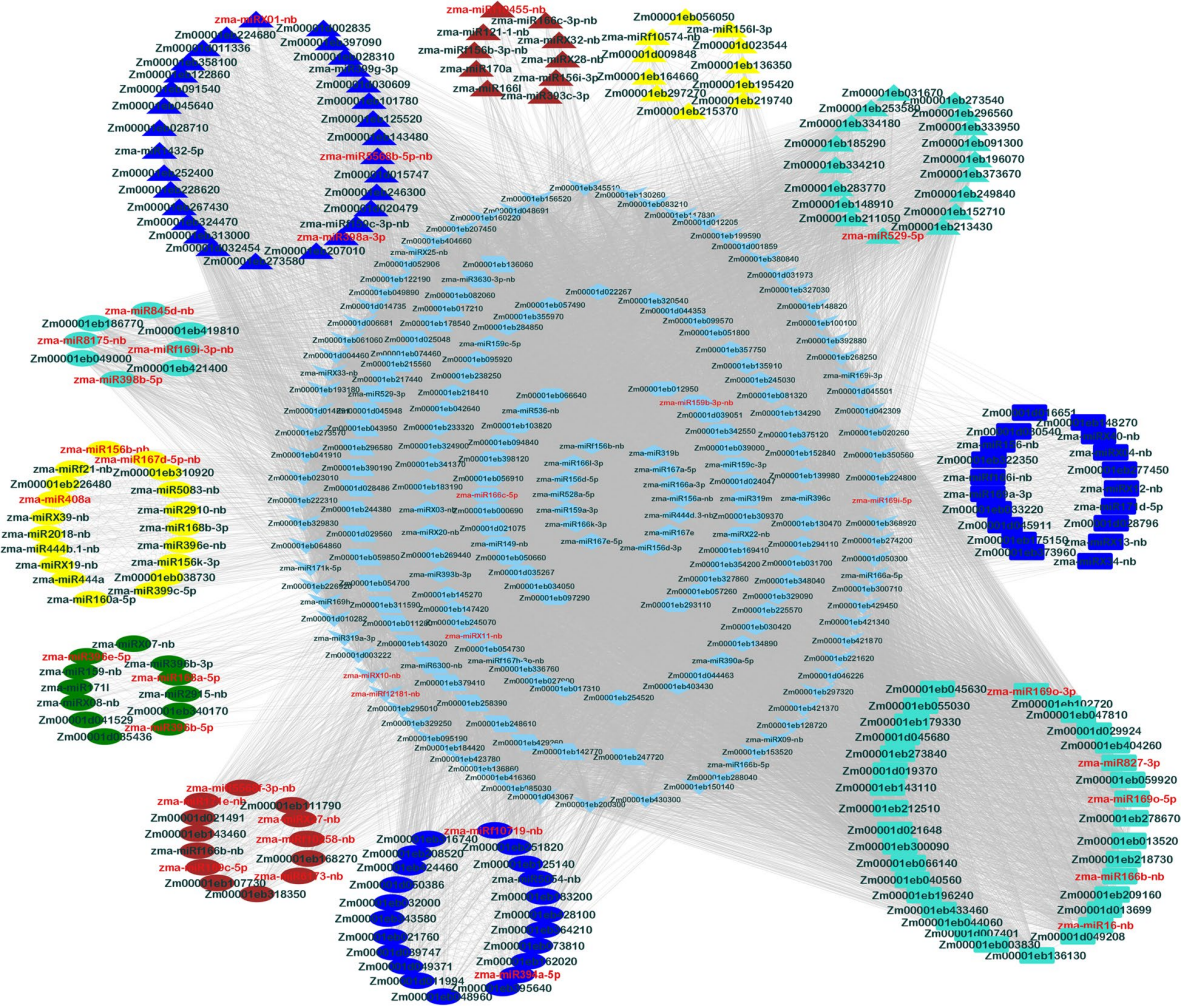
Cystoscope network depicting different modules of coexpression network from three different maize genotypes. The nodes highlighted in red were miRNAs having significant module membership and intramodular connectivity metrics. Five, four, and two significant modules were identified in MI82, TZAR102 and Va35 respectively that are assigned with different node shapes. Ellipse – MI82 only; Triangle – TZAR102 only; Rectangle – Va35 only; Diamond -MI82_N_TZAR102; Hexagon – MI82_N_Va35; Parallelogram – TZAR102_N_Va35; V = MI82_N_TZAR102_N_Va35

Weighted coexpression network analysis using the differentially expressing genes and their targets based on their expression values in all three genotypes identified three significant modules at ≥ 0.2 significance (Supplementary Table S16; Fig. 9). Based on -0.8 ≤ kME ≥ 0.8 cut off at and gene significance at ≥ 0.2, 54, 16, and 13 miRNAs/genes (nodes) belonged to Turquoise, Blue, and Black modules, respectively. Top nodes with highest number of connections within each of these modules with ≥ 0.2 weight revealed that zma-miR169h, zma-miRX33-nb, zma-miR166a-5p, zma-miR166b-5p, zma-miRX09-nb, zma-miRX20-nb, zma-miRX25-nb, and zma-miRX03-nb with maximum number of interactions (53) as hub miRNAs in the Turquoise module. The connections among the nodes in other modules were not strong.

Genotype-specific coexpression networks depicted four significant modules (Turquoise, Yellow, Brown, and Blue) for the resistant cultivar TZAR102 while only two modules were significant for the susceptible cultivar Va35 (Supplementary Table S17). Based on the number of connections (≥ 90% of the nodes) at ≥ 0.2 strength, zma-miR529-5p (92) in Turquoise, zma-miRf10455-nb (22) in Brown, and zma-miR5568b-5p-nb (81), zma-miRX01-nb (80), and zma-miR398a-3p (76) in Blue modules were considered as hubs. On the other hand, five significant modules, Yellow, Turquoise, Green, Brown, Blue, and Red were identified for MI-82 with 31, 88, 21, 47, 58, and 4 genes, respectively. The miRNAs, zma-miR167d-5p-nb (30), zma-miR408 (29), zma-miR156b-nb (28) and zma-miR168b-3p (28) in Yellow module, zma-miRf169i-3p-nb (87), zma-miR8175-nb (87), zma-miR845d-nb (85), and zma-miR398b-5p (84) in Turquoise, zma-miR396b-5p (20), zma-miR396e-5p (20), and zma-miR168a-5p (19) in Green, zma-miRf10258-nb, zma-miRX11-nb, zma-miRf12181-nb, zma-miR166c-5p, zma-miRX10-nb, zma-miRX27-nb, zma-miR169i-5p, zma-miR159b-3p-nb, zma-miR171e-nb, zma-miR6173-nb, and zma-miR5568f-3p-nb, all with 46 connections and zma-miR169c-5p with 45 connections in Brown, zma-miR394a-5p and zma-miRf10719-nb, both with 57 connections in Blue module qualified as the hub nodes in MI82. The hub miRNAs in the susceptible variety Va35 were zma-miR169o-5p, zma-miR827-3p, zma-miR169o-3p, zma-miR166b-nb, and zma-miR16-nb, all with 151 connections in Turquoise, and zma-miR169a-3p, zma-miRf166i-nb, zma-miRX40-nb, zma-miRX34-nb, zma-miRX13-nb, zma-miR171d-5p, zma-miR156-nb, zma-miRX04-nb, and zma-miRX12-nb with 38 connections all.

The miRNA hubs in resistance modules (TZAR102 and MI82) were connected to the genes that are related predominantly to transcription factors and protein kinases (Supplementary Table S18.1). Of the 34 hubs, nine had a node in the module connected to their target gene (Supplementary Table S18.2). Therefore, these nine miRNA-mRNA pairs were considered to have important roles in resistance/susceptible response of maize to *Aspergillus flavus*. The module of miRNA zma-miR398a-3p targeting F-box protein SKIP5 (Zm00001eb329830) in TZAR102 and zma-miR394a-5p targeting another F-box protein 6 (Zm00001d011994) in MI82 were considered important candidates for downstream studies. Similarly, miRNAs such as zma-miRf10719-nb, zma-miR396e-5p, and zma-miR396b-5p with Zm00001eb329250, Zm00001eb139980, Zm00001eb278670, and Zm00001eb139980 genes as their co-expressing partners/targets are worth pursuing for their involvement in quantitative resistance against *A. flavus*.

## Discussion

Aflatoxin contamination is a major worldwide problem, yet the underlying molecular mechanisms of maize resistance to *Aspergillus flavus* remains elusive. Identification of some maize genotypes with resistance to *A. flavus* infection and aflatoxin contamination than others in the germplasm implies that breeding for resistance can be an effective strategy for lowering the risk of aflatoxin contamination in maize (Brown et al. 1999). Plants have a multi-layered defense system against pathogens, including basal resistance, hypersensitive response (HR), and host-induced gene silencing (Prasad et al. 2023). Identification of regulatory elements can help uncover the machineries at the post-transcriptional level that are involved in the resistance/susceptibility-associated mechanisms. Although metabolites have traditionally been used as biomarkers for aflatoxin resistance (Wild and Turner 2002; Lau et al. 2006; Kensler et al. 2011), small non-coding RNAs such as miRNAs were presented as an additional avenue for biomarkers development that could be cheap and easily quantifiable with next generation sequencing (Lopez et al. 2015).

Reports are available on possible involvement of miRNAs and their target genes in maize response to biotic stresses such as *Fusarium* ear rot (Zhou et al. 2020) and abiotic stresses such as drought (Tang et al. 2022). But this is the first ever comprehensive study on genotype and time-scale miRNA profiles following *A. flavus* infection in maize kernels. In this high-throughput sequencing study, we monitored the miRNA profiles in three maize genotypes with differing response to *A. flavus* at three distinct time points. The results presented in the present study suggest implications of miRNAs as regulatory factors in maize for aflatoxin resistance. Differential expressions of the miRNAs in all three genotypes suggested that miRNAs are involved in both resistance and susceptibility response of maize to the fungal infection. The induction/suppression of miRNAs belonging to different miRNA families indicates functional diversification of miRNAs regulating expression of genes in various biological pathways involved in *A. flavus* infection reaction. The expression of the enriched GO terms or pathways of the upregulated miRNA targets were suppressed, and the opposite was true for downregulated miRNA targets. The expression of the miRNAs and their corresponding target genes mostly were coherent, where miRNA upregulation led to less mRNA abundance, although some incoherent or semi-coherent patterns in their expression were observed (Supplementary Figure S10).

Very few miRNAs showed apparently differential expression after 8 h of *A. flavus* inoculation (8HPI). While it is known that most transcriptional changes happen early in the plant-pathogen interaction, it is hypothesized that such transcriptional regulation did not happen immediately at an early stage after *A. flavus* inoculation. It is possible that in the absence of a typical gene-for-gene interaction with *A. flavus* and the host crop, the plant (post)transcriptional response unravels at later time points when the pathogen attempts to establish itself for the infection to occur/progress. The higher number of miRNAs differentially expressed in all three lines at 3 d (3DPI) and 7 d post inoculation (7DPI) relative to 8HMI testified this proposition.

The miR169i was commonly upregulated in all three genotypes after 3 days post inoculation targets eight transcription factors (Zm00001eb005690, Zm00001eb019780, Zm00001eb032000, Zm00001eb050320, Zm00001eb108930, Zm00001eb218610, Zm00001eb256650, and Zm00001eb327230), which are related to CCAAT-binding factor (CBF) complex/ nuclear factor Y (NF-Y). The CBF complex has been implicated in various cellular processes, including iron homeostasis by regulation of genes that encode iron uptake transporters so inhibition of the CBF complex can potentially impact bioavailability of iron in host cells or deficiency in invading fungal cells (Gsaller et al. 2014). Upregulation of miR169i disrupts CBF complex activity followed by depletion of iron availability by inhibiting its uptake (Gsaller et al. 2014). These results suggest that CBF may have a functional role in general short-term defense of plants against *A. flavus* establishment by limiting nutrient availability, especially iron. The overexpression of nine novel candidate miRNAs and four dominant families suggests that these families may play a vital role in biotic stress alleviation in maize kernels. In general, MI82 showed the highest number of DEMs at any given stage with a total of 64 DEMs (36 upregulated and 28 downregulated) compared to TZAR102 and Va35. This explains the possible interplay of various biological mechanisms regulated by multiple miRNAs with possible minor effects contributing to the moderate resistance of MI82. When compared against the susceptible variety Va35, miR393 that showed upregulation in MI82 but downregulation in TZAR102, contributes to pathogen-associated molecular patterns (PAMPs) induced immunity (PTI) in Arabidopsis by negatively regulating transport inhibitor response 1 protein and auxin signaling pathways (Navarro et al. 2006). This suggested that PAMPs-based resistance could be an important mechanism underlying resistance against the invading pathogen in MI82.

The primary goal of this study was to elucidate post-transcriptional mechanisms operating in the resistant variety, and hence we focused the discussion on the miRNAs whose accumulation was significantly increased or decreased in TZAR102 as compared to the susceptible variety Va35 and the moderately resistant variety MI82. The most highly upregulated zma-miRX31-nb in TZAR102 targets the expansin-B1-like gene. Expression of expansins, which are non-enzymatic cell-wall remodeling proteins, can affect plant resistance or susceptibility to infection either by direct modification of the cell wall stiffness, surface tension, or barrier properties or by triggering plant immune responses (Marowa et al. 2016; Narváez-Barragán et al. 2020). The miRNAs, zma-miRX01-nb, zma-miRf167e-3p-nb, and zma-miR11970-5p that were upregulated specifically in TZAR102 at 3 d and 7d after inoculation targeted serine threonine protein kinase, which was highly (3.8-fold) downregulated and an upregulated phosphoenolpyruvate carboxylase (PEPC) gene. Comparative proteomics and transcriptomics studies with maize kernels have suggested potential role of serine-threonine protein kinase in resistance to *A. flavus* infection and aflatoxin production (Luo et al. 2011; Chen et al. 2015). While the function of PEPC as a key enzyme in C4 photosynthesis has been established in plant stress resistance, its role in *A. flavus* is not known. However, its role in regulation of pH and stomatal opening implies its possible implication in the pathogen establishment and proliferation. On the other hand, zma-miR6478-nb was downregulated only in TZAR102 at the same time points where its target WD-domain beta-G protein also manifested downregulation after 7d of inoculation with the fungus. Heteromeric beta-G proteins are known transducers of receptor signaling that have functional roles in PAMP receptors controlling basal immunity against pathogens, and therefore was proposed as a target for genetic modification in maize with potentially optimized trade-off between growth and defense signaling (Wu et al. 2019). The miRNAs belonging to the family miR159 and miR171 were downregulated in TZAR102 whereas they were identified as hubs in MI82. Similarly, members of miR166 family that were upregulated in both TZAR102 and MI82 relative to Va35 targeted HD-ZIP transcription factor, which expectedly showed downregulation upon fungal infection.

In maize, several transcription factor gene families are known to be involved in various developmental processes, metabolic pathways, and (a)biotic stress responses, including *A. flavus* resistance (Chen et al. 2015; Hawkins et al. 2018; Musungu et al. 2020; Liu et al. 2021; Baisakh et al. 2023). The results suggested that the regulatory miRNA-transcription factor pairs including miR159-MYB, miR171-GRAS, miR166-HD-ZIP, miR169-AP2, and miRNA156-SBP are potential targets for genetic manipulation of maize for *A. flavus* resistance. Of special consideration is the Zma-miR156-squamosa promoter binding protein (SBP), Zma-miR398-F-box, and Zma-miR394-F-box combinations where members of miR156, miR398, and miR394 family were identified as hubs with SBP and F-box proteins as coexpression partner and targets, respectively in the resistance-associated modules. Members of the miRNA156 family, zma-miRf156e-3p-nb and zma-miR156b-3p were highly downregulated in TZAR102 and their targets flavin-containing monooxygenase (FMO) and Phox-domain protein kinase, respectively also showed slight downregulation in their transcript abundance. FMOs were found to be critical in systemic acquired resistance in plants by triggering cell death likely through synthesis of a metabolite that is required for the signal transduction or amplification during early phases of SAR establishment (Mishina and Zeir 2006; Krönauer and Lahaye 2021). Similarly, Phox-domain protein kinases are recognized as new players of stress resistance mechanism including their roles in resistance against fungal pathogen *Fusarium graminearum* via phosphoinositide signaling (Lou et al. 2021). On the other hand, zma-miR156b-nb showed upregulation as a hub in MI82 with the expression of its target gene SBP being highly repressed following fungal infection. While SBPs regulate the transcription of downstream genes by binding of the SBP domain to GTAC core motif (Yang et al. 2008) and thus function in plant growth and development and stress responses, they are also degraded by miRNAs with the miRNA responsive element downstream of the SBP domain (Gandikota et al. 2007). SBP was differentially expressed in maize upon infection with *A. flavus* (Hawkins et al. 2018) and the specific miR156-SBP pair was identified as one of the two most important combinations with differential expression in peanut in response to *A. flavus* (Zhao et al. 2020). F-box protein SKIP5 was computationally predicted and validated as a target in the degradome. Zma-miR398a-3P and Zma-miR394a-5P were negatively regulated in TZAR102 and MI82 while the F-box protein targets were upregulated in TZAR102 and slightly downregulated in both TZAR102 and MI82 in response to *A. flavus* infection. The involvement of miR394 in disease resistance was shown by its action as a negative regulator of gray mold resistance in tomato and Arabidopsis (Jin and Wu 2015; Tian et al. 2018) where the overexpression of miR394 enhanced disease susceptibility by targeting an F-box protein (Tian et al. 2018). Upregulation of miR398 was reported in tomato upon infection with *Bemicia tabaci* (Wang et al. 2018). The differential expression of miR398 and target pair could be related to the differential resistance response of the resistant and susceptible genotypes (Rabuma et al. 2021). The target genes encoding F-box proteins are the component of SCF (for SKP1/CUL1/F-box) complexes that function as ubiquitin E3 ligases involved in multiple biological mechanisms. Several members of the F-box gene superfamily are known to participate in pathogen response (Lechner et al. 2006) and downregulation of F-box protein could compromise hypersensitive response mediated resistance by regulating cell death during pathogen recognition (van Ooijen et al. 2007; van den Burg et al. 2008). The involvement of F-box protein in PR proteins-mediated *A. flavus* resistance response was demonstrated either by its downregulation in maize PRms RNAi lines (Majumdar et al. 2017) or upregulation in peanuts during the late stages of infection by the fungus (Bhatnagar-Mathur et al. 2021).

**Fig. 10:**
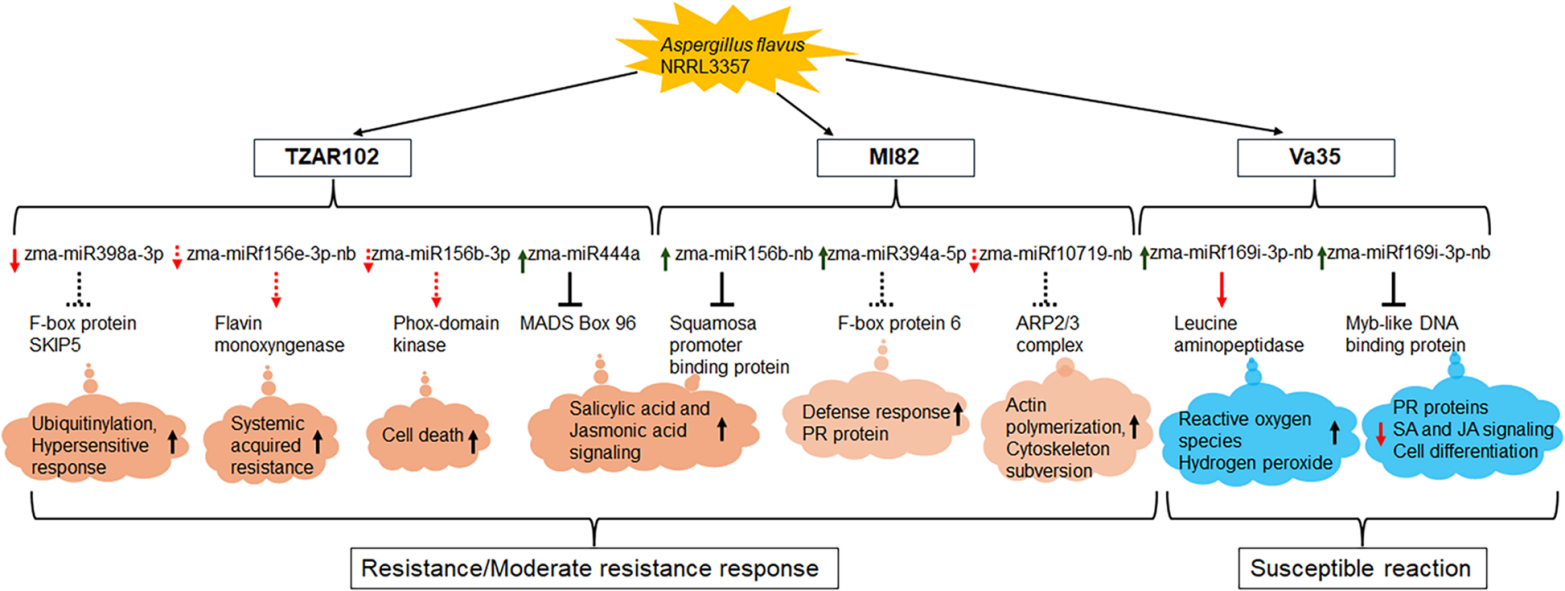
A schematic illustration of miRNAs and their corresponding targets involved in various mechanisms (metabolic and signaling pathways) with relevance in resistance, moderate resistance, and susceptibility of maize kernels of three genotypes (TZAR102, MI82, and Va35) in response to *Aspergillus fungus* infection. Arrows left to the miRNAs indicate their up (green) and down (red) regulation. Blocked lines and arrows connecting miRNAs to corresponding gene targets represent suppression and induction of their expression, respectively. Solid or dotted lines/arrows represent the strength of the response. Up (green) and downward (red) arrows for mechanisms indicate their induction and repression, respectively

Collectively, our research results presented here integrated the expression of miRNAs and their targets (Castano-Duque et al. 2021) in maize and identified a promising number of known and expression-validated novel miRNAs and their coexpression/target partners that can potentially be utilized as biomarkers for resistance against *A. flavus* growth and/or aflatoxin production (Fig. 10). However, while most of the targets of the differentially expressed miRNAs identified in this study also had perturbed expression upon fungus infection, it is important to note the limitation of the results as not all possible miRNA-target pairs were identified in the network due to the threshold set for selection of node/hub, which could still be important candidates for fungal resistance. Nevertheless, this is the first comprehensive analysis of the posttranscriptional regulation in maize utilizing both miRNA and transcriptomic data of maize-*A. flavus* system. Detail functional studies of the miRNA hub-target pairs identified in this study through gene manipulation (gene engineering or editing) and/or introgression would enhance our understanding of the molecular intricacies involved in host (maize) resistance response against the opportunist *A. flavus* fungus. The validated biomarkers would complement or enhance the accuracy of current methods of testing for aflatoxin contamination in maize and potentially other affected agricultural products, which will indirectly impact global economy and health.

### Supplementary Information

**Additional file 1:Supplementary Figure S1.** Length distribution of the small RNAs from the three maize lines, TZAR102, MI82, and Va35. T = TZAR102, M = MI82, V = Va35, 8HMI = 8 h post mock-inoculation, 8 HPI = 8 h post inoculation with *Aspergillus flavu*s, 3DPI = 3 d post inoculation with *A. flavu*s, 7DPI = 7 d post inoculation with *A. flavu*s

**Additional file 2:Supplementary Figure S2.** Venn diagram showing known miRNAs common between and unique to the three maize lines, TZAR102, MI82, and Va35.

**Additional file 3:Supplementary Figure S3.** Pie chart showing abundance of known miRNA families identified from TZAR102, MI82, and Va35 genotypes of maize.

**Additional file 4:Supplementary Figure S4.** Venn diagram showing putative novel miRNAs common between and unique to the three maize lines, TZAR102, MI82, and Va35.

**Additional file 5:Supplementary Figure S5.** Secondary hairpin structure of two representative known and novel miRNAs identified in maize with or without inoculation with *Aspergillus flavus*.

**Additional file 6:Supplementary Figure S6.** Length distribution (a), overall nucleotide bias (b), nucleotide bias by length (c) of 316 miRNAs identified from maize genotypes.

**Additional file 7:Supplementary Figure S7:** Number of up (blue bars) and down (orange bars) regulated differentially expressed miRNAs in different studied conditions. Bar names a to m represents their respective studied combinations. Number of DEMs were labeled on top/bottom of the bars.

**Additional file 8:Supplementary Figure S8.** Circos diagram showing distribution of 316 miRNAs shown as black bands on the outer circle of maize genome with 1MB window size on the karyotype. All 41 putative novel miRNAs are labeled in blue. The second and third outer circles represent the target genes on positive and negative strands, respectively. The miRNAs and corresponding target genes are interconnected radially by different colored lines in the center.

**Additional file 9:Supplementary Figure S9.** Target plots (t-plots) for important miRNA targets confirmed by degradome sequencing.

**Additional file 10:Supplementary Figure S10.** Reverse transcription PCR showing expression pattern of miRNAs (a) and corresponding targets (b) in maize genotypes TZAR102, MI82, and Va35 at 8 h post mock inoculation, 8 h, 3 d, and 7d post inoculation with *Aspergillus flavus*. T = TZAR102, M = MI82, V = Va35, 8HMI = 8 h post mock-inoculation, 8 HPI = 8 h post inoculation with *Aspergillus flavu*s, 3DPI = 3 d post inoculation with *A. flavu*s, 7DPI = 7 d post inoculation with *A. flavu*s

**Additional file 11:Supplementary Table S1.** Sequences of the primers used for reverse-transcription PCR of the differentially expressed miRNAs including putative novel miRNAs.

**Additional file 12:Supplementary Table S2.** Sequences of the primers used for reverse transcription PCR of the target genes of the differentially expressed miRNAs.

**Additional file 13:Supplementary Table S3.** Descriptive pre- and post-bioinformatic statistics of the small RNA data from TZAR102, MI82, and Va35 lines of maize sequenced at 8 h post mock inoculation and 8h, 3d, and 7d post inoculation with *Aspergillus flavus*.

**Additional file 14:Supplementary Table S4.** List of 171 miRNA isoforms identified from TZAR102, MI82, and Va35 lines of maize in present study but not previously reported in maize.

**Additional file 15:Supplementary Table S5.** List of 275k known miRNAs that belonged to 114 known miRNA families.

**Additional file 16:Supplementary Table S6.** Diversity and abundance of known miRNA families identified from TZAR102, MI82, and Va35 genotypes of maize.

**Additional file 17:Supplementary Table S7.** Detail statistics of 41 putative novel miRNAs identified in maize lines with or without inoculation with *Aspergillus flavus*.

**Additional file 18:Supplementary Table S8.** Sequence, physical location, and length of the 316 miRNAs identified from maize lines with or without inoculation with *Aspergillus flavus*.

**Additional file 19:Supplementary Table S9.** Differentially expressed miRNAs in maize treated with *Aspergillus flavus* relative to mock-inoculated control.

**Additional file 20:Supplementary Table S10.** Differentially expressed miRNAs in TAR102 relative to Va35 regardless of treatment and time points.

A**dditional file 21:Supplementary Table S11.** Differentially expressed miRNAs in MI82 relative to Va35 regardless of treatment and time points.

**Additional file 22:Supplementary Table S12.** Differentially expressed miRNAs in TAR102 relative to MI82 regardless of treatment and time points.

**Additional file 23:Supplementary Table S13. **1. Target genes of 311 out of 316 unique miRNAs identified in maize lines with or without inoculation with *Aspergillus flavus*. S13.1 – number and family; S13.2. annotation and sequence information, S13.3. number of miRNAs potentially targeting the same target gene.

**Additional file 24:Supplementary Table S14.** Target genes of the unique miRNAs identified from the publicly available maize degradome libraries.

**Additional file 25:Supplementary Table S15.** Gene ontology (GO) terms enrichment for genes upregulated (S15.1, S15.3, and S15.5) and downregulated (S15.2, S15.4, and S15.6) in biological process, molecular function, and cellular component, respectively.

**Additional file 26:Supplementary Table S16.** Coexpression network modules with significant miRNAs and target genes across all three (TZAR102, MI82, and Va35) genotypes of maize.

**Additional file 27:Supplementary Table S17.** Significant coexpresison network modules represented in TZAR102, MI81, and Va35 genotypes of maize.

**Additional file 28:Supplementary Table S18.** Hub genes in different modules specific to three genotypes TZA102, MI82, and Va35 (S18.1) and information on their network partners and targets (S18.2).

## Data Availability

All data analyzed during this study are included in this published article [and its supplementary information files]. All small RNA raw sequence data reported in this study have been submitted to the NCBI SRA database and are available under BioProject accesssion ID PRJNA1060245 with BioSamples accessions (SAMN 39222440 – SAMN 39222475).

## Abbreviations

AF: Aflatoxin
AGO: Argonaute
AP2: Apetala
bp: Base pair
CBF: CCAAT-binding factor
D: Day
DEM: Differentially expressed miRNAs
DPI: Days post inoculation
FC: Fold change
FMO: Flavin-containing monooxygenase
FPKM: Fragments per kilobase of transcript per million mapped reads
GO: Gene ontology
GRAS: Gibberellic acid insensitive, repressor of GAI, and scarecrow
H: Hour
HMI: Hours after mock-inoculation
HD-ZIP: Homeodomain leucine zipper
HPI: Hours post inoculation
KSA: Kernel screening assay
HR: Hypersensitive response
lncRNA: Long non-coding RNA
μl: Microliter
miRNA: MicroRNA
MYB: Myeloblastosis
NAC: NAM, ATAF1/2, and CUC2
ng: Nanogram
NF-Y: Nuclear factor Y
nt: Nucleotide
pM: Picomole
PMRD: Plant miRNA database
SBP: Squamosa promoter binding protein
PAMP: Pathogen-associated molecular pattern
PTI: Pattern-triggered immunity
rRNA: Ribosomal RNA
RT-PCR: Reverse transcription polymerase chain reaction
SAR: Systemic acquired resistance
SKIP5: SKP1/ASK-interacting protein 5
snRNA: Small noncoding RNA
snoRNA: Small nucleolar RNA
tRNA: Transfer RNA
tasiRNA: Trans-acting small interfering RNA
TAE: Tris acetate EDTA
TF: Transcription factor
