# Supplementary figures and images for: MicroRNA (miRNA) profiling of maize genotypes with differential response to Aspergillus flavus implies zma-miR156–squamosa promoter binding protein (SBP) and zma-miR398/zma-miR394–F -box combinations involved in resistance mechanisms
Source: Stress Biol. 2024 May 10;4(1):26. doi: 10.1007/s44154-024-00158-w (PMC11087424; doi:10.1007/s44154-024-00158-w)

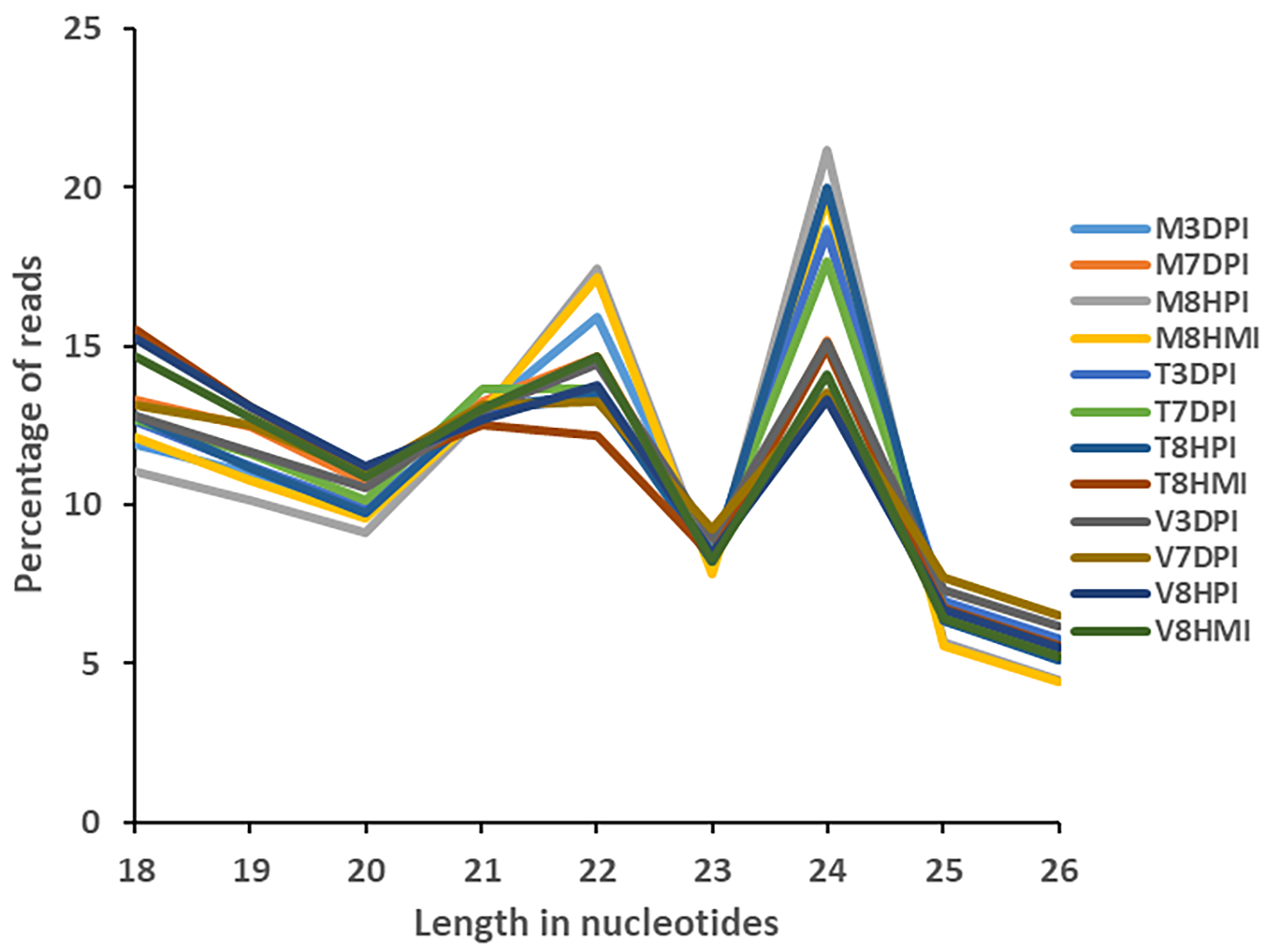

Supplement: Supplementary file 1 — Additional file 1:Supplementary Figure S1. Length distribution of the small RNAs from the three maize lines, TZAR102, MI82, and Va35. T = TZAR102, M = MI82, V = Va35, 8HMI = 8 h post mock-inoculation, 8 HPI = 8 h post inoculation with Aspergillus flavus, 3DPI = 3 d post inoculation with A. flavus, 7DPI = 7 d post inoculation with A. flavus [file 44154_2024_158_MOESM1_ESM.png]

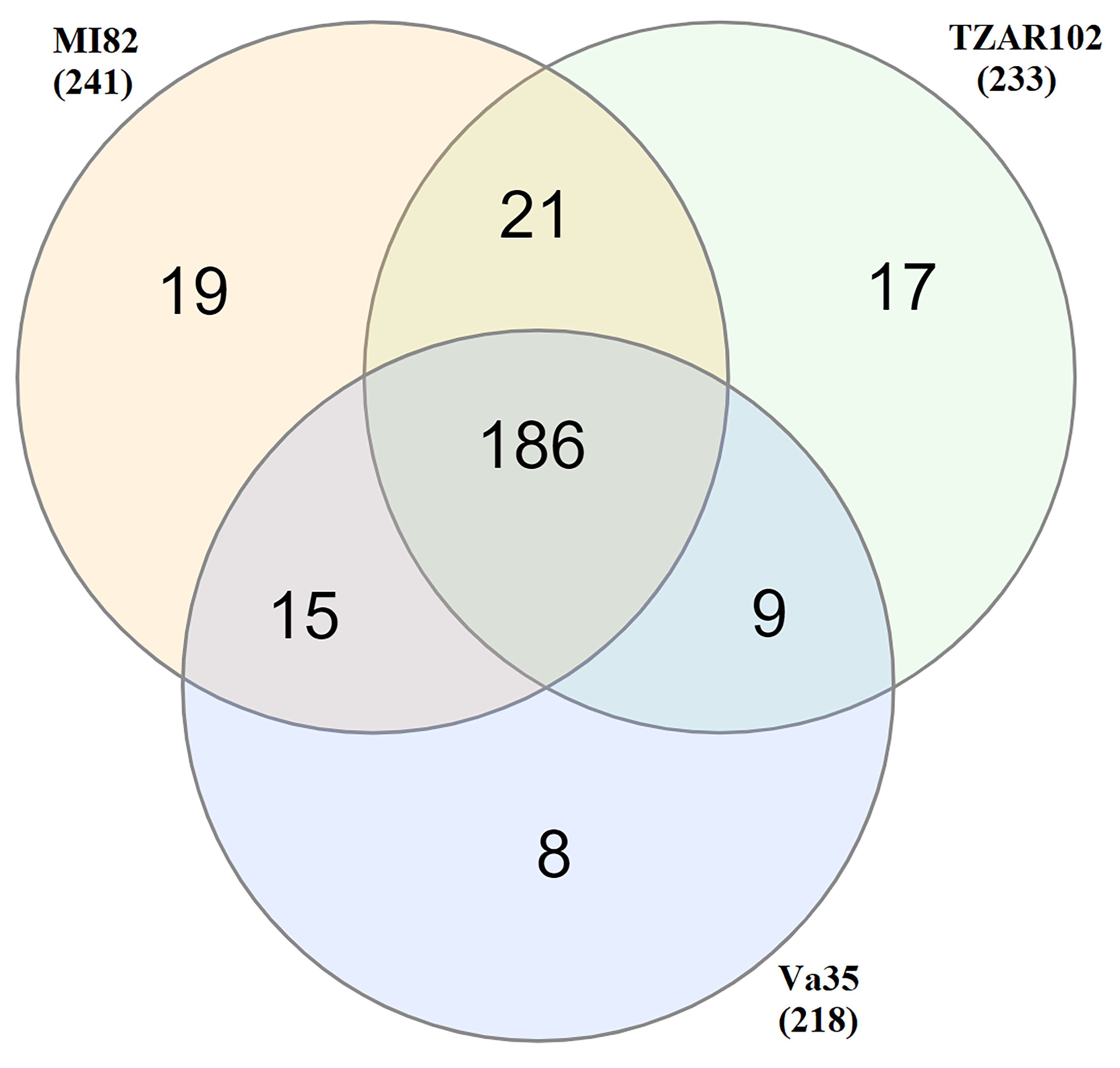

Supplement: Supplementary file 2 — Additional file 2:Supplementary Figure S2. Venn diagram showing known miRNAs common between and unique to the three maize lines, TZAR102, MI82, and Va35. [file 44154_2024_158_MOESM2_ESM.png]

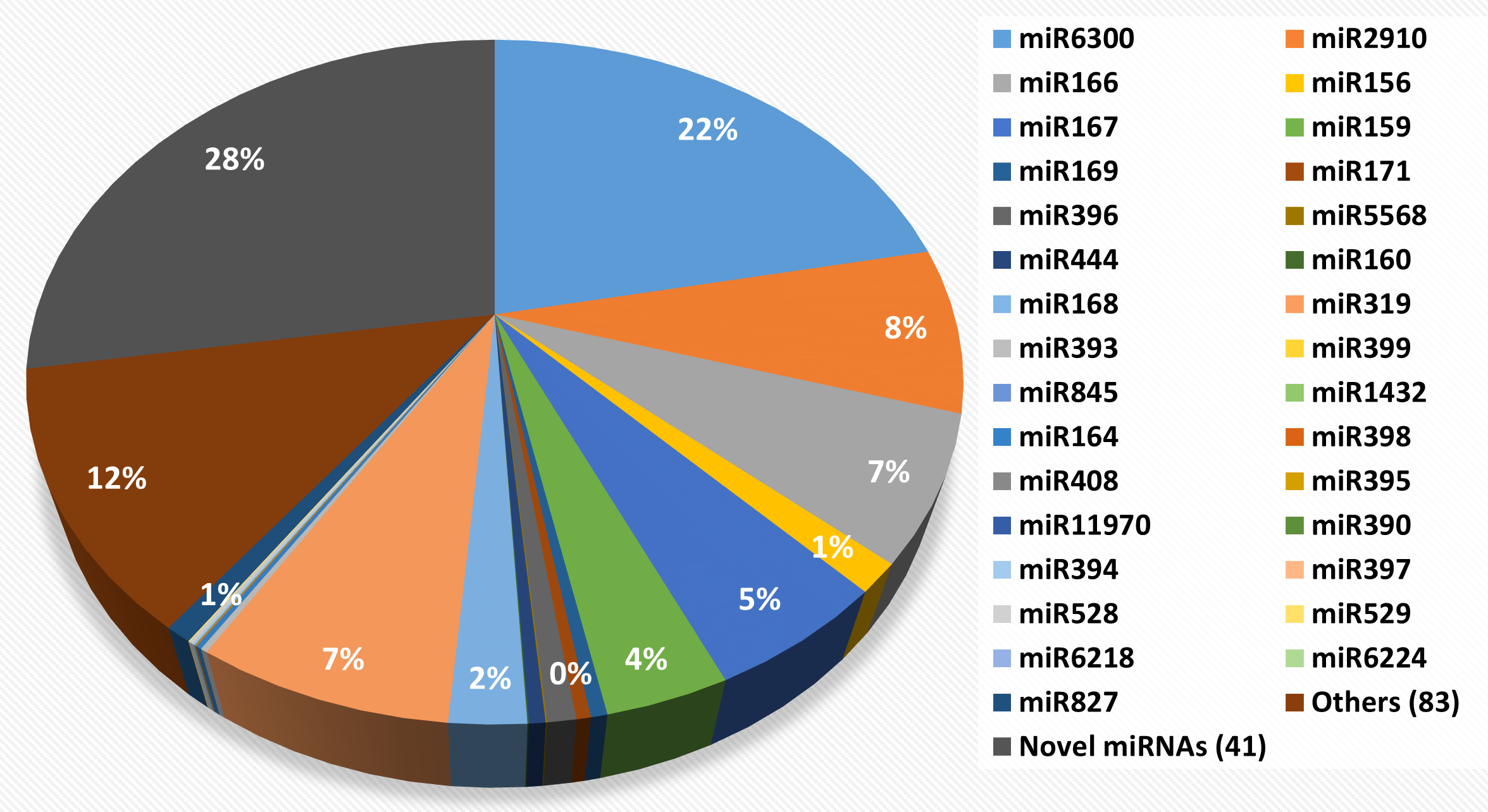

Supplement: Supplementary file 3 — Additional file 3:Supplementary Figure S3. Pie chart showing abundance of known miRNA families identified from TZAR102, MI82, and Va35 genotypes of maize. [file 44154_2024_158_MOESM3_ESM.png]

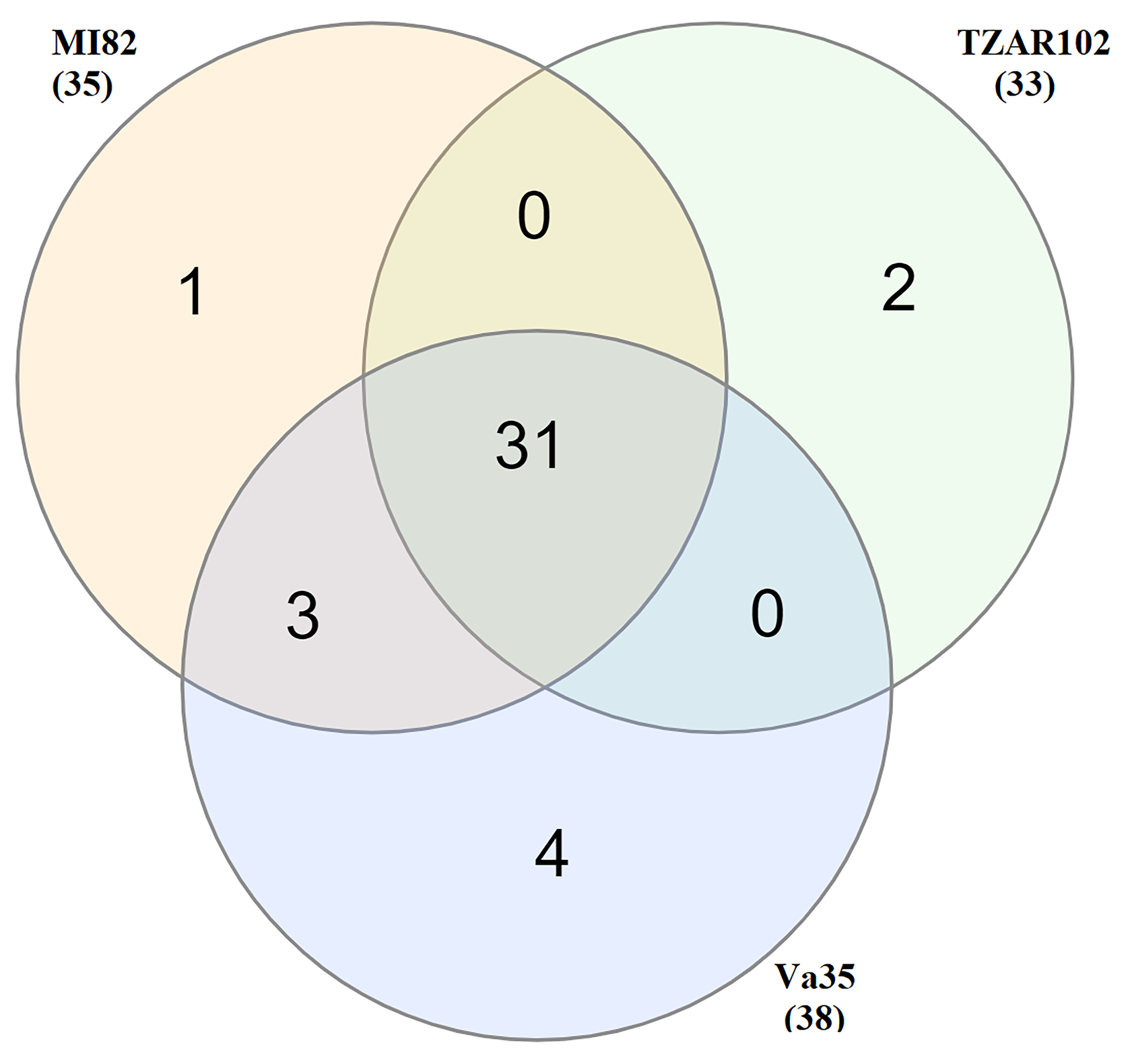

Supplement: Supplementary file 4 — Additional file 4:Supplementary Figure S4. Venn diagram showing putative novel miRNAs common between and unique to the three maize lines, TZAR102, MI82, and Va35. [file 44154_2024_158_MOESM4_ESM.png]

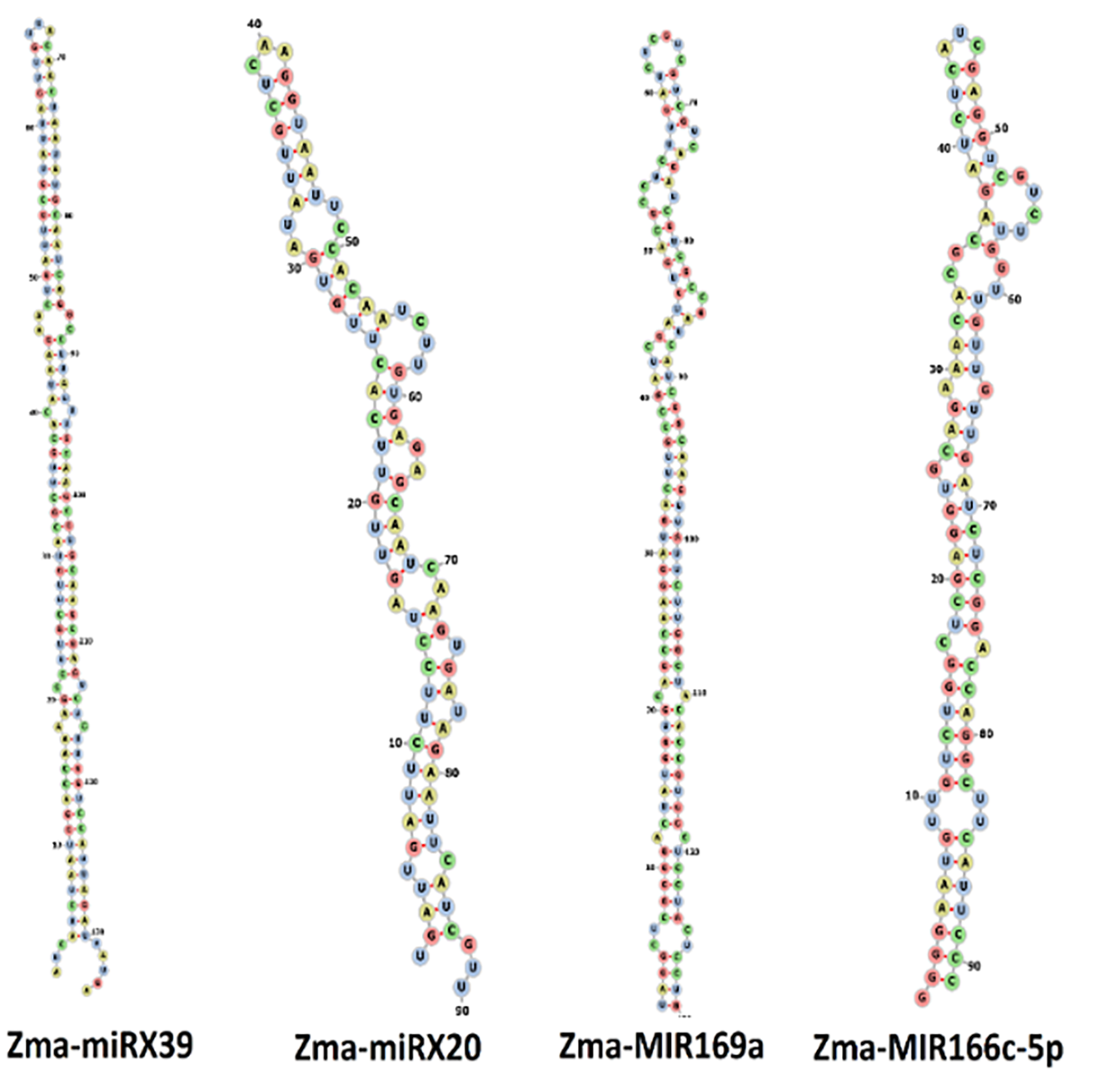

Supplement: Supplementary file 5 — Additional file 5:Supplementary Figure S5. Secondary hairpin structure of two representative known and novel miRNAs identified in maize with or without inoculation with Aspergillus flavus. [file 44154_2024_158_MOESM5_ESM.png]

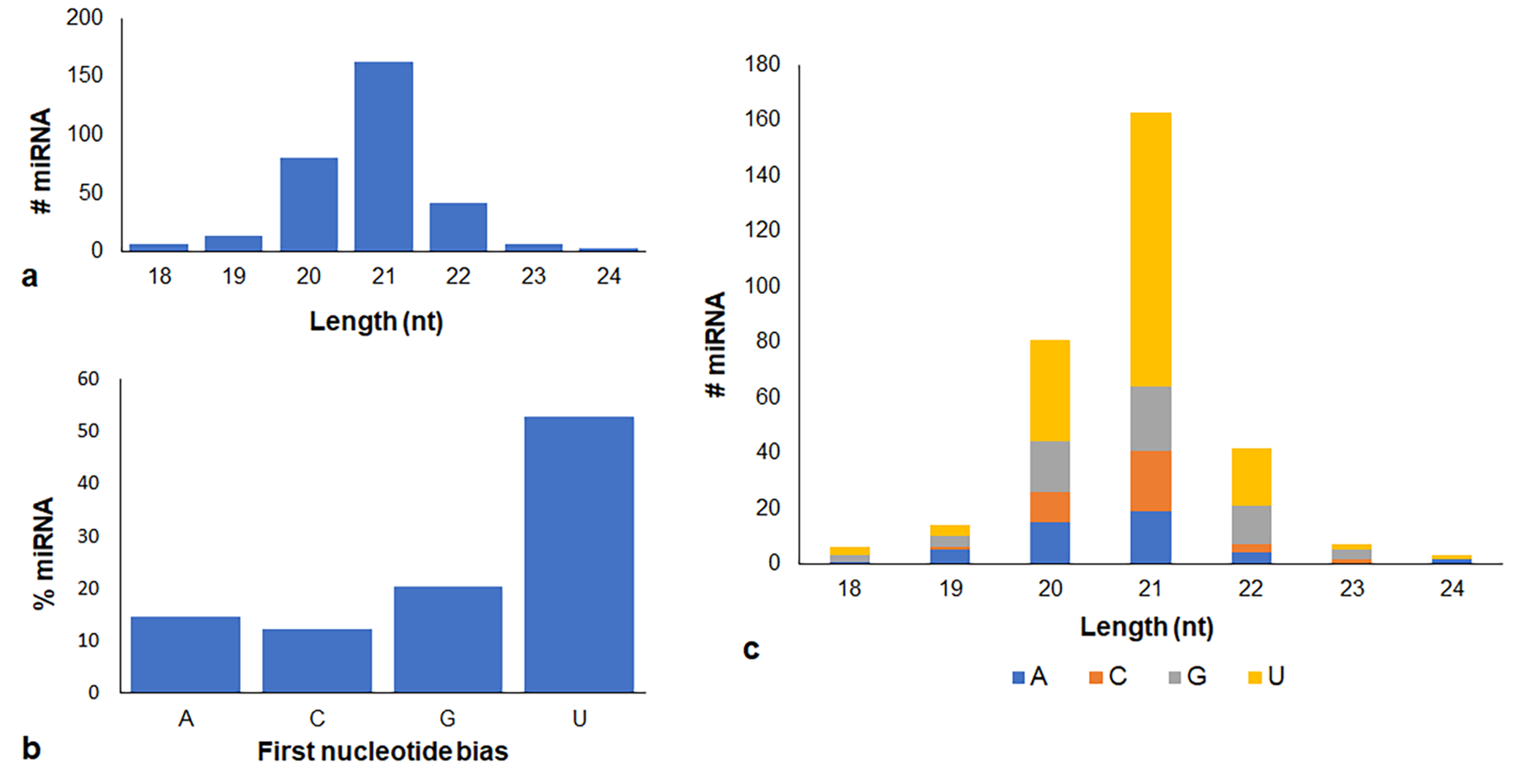

Supplement: Supplementary file 6 — Additional file 6:Supplementary Figure S6. Length distribution (a), overall nucleotide bias (b), nucleotide bias by length (c) of 316 miRNAs identified from maize genotypes. [file 44154_2024_158_MOESM6_ESM.png]

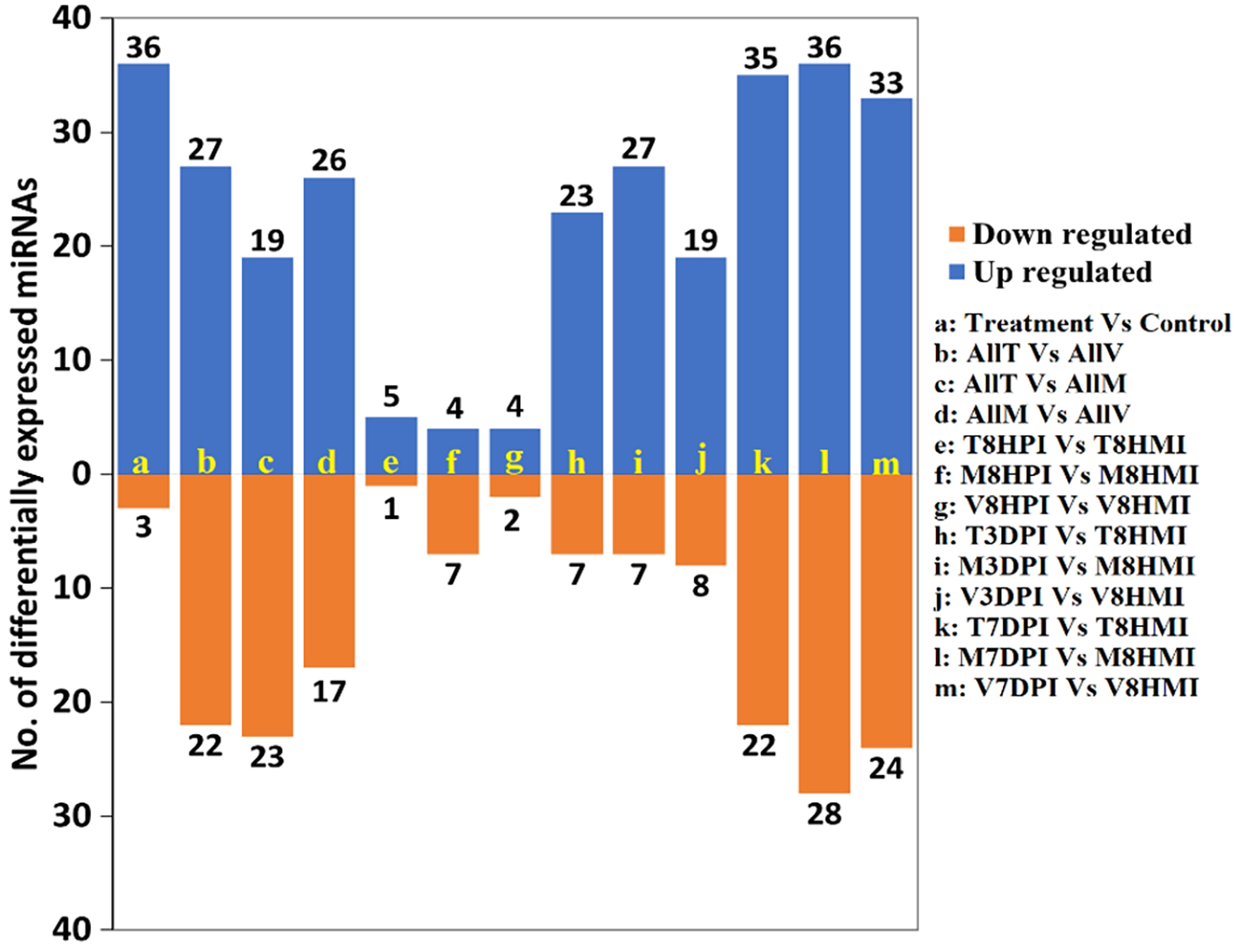

Supplement: Supplementary file 7 — Additional file 7:Supplementary Figure S7: Number of up (blue bars) and down (orange bars) regulated differentially expressed miRNAs in different studied conditions. Bar names a to m represents their respective studied combinations. Number of DEMs were labeled on top/bottom of the bars. [file 44154_2024_158_MOESM7_ESM.png]

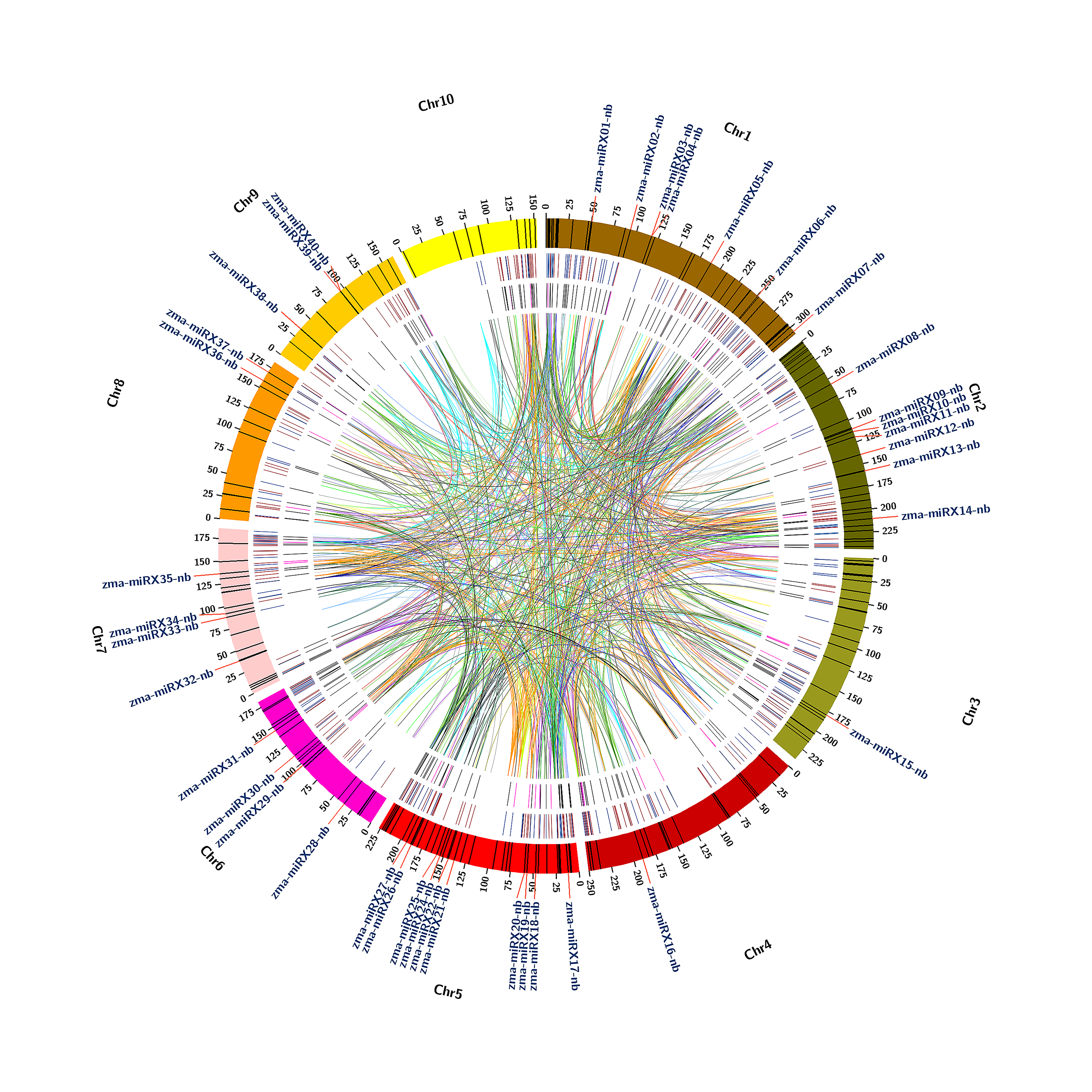

Supplement: Supplementary file 8 — Additional file 8:Supplementary Figure S8. Circos diagram showing distribution of 316 miRNAs shown as black bands on the outer circle of maize genome with 1MB window size on the karyotype. All 41 putative novel miRNAs are labeled in blue. The second and third outer circles represent the target genes on positive and negative strands, respectively. The miRNAs and corresponding target genes are interconnected radially by different colored lines in the center. [file 44154_2024_158_MOESM8_ESM.png]

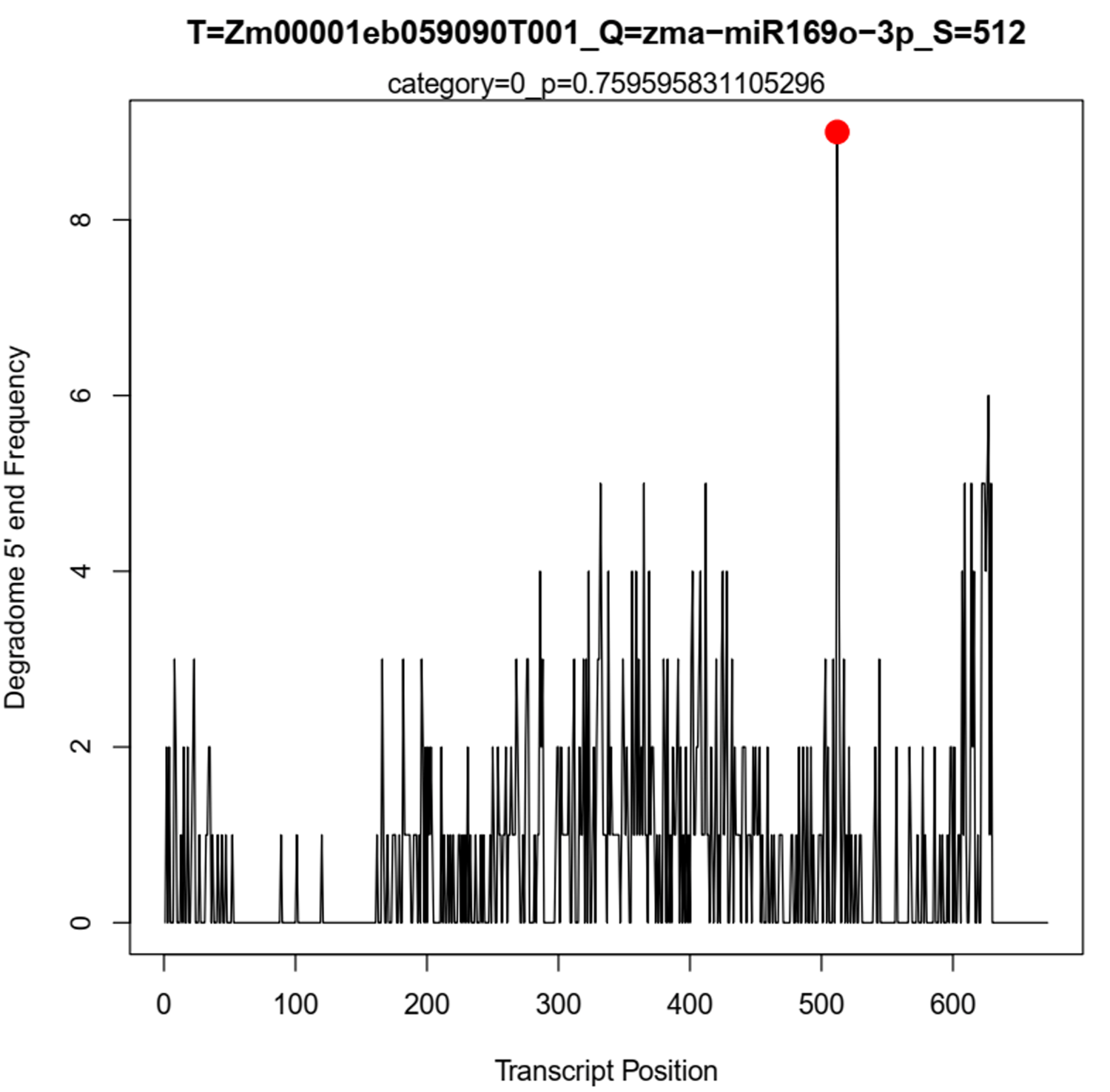

Supplement: Supplementary file 9 — Additional file 9:Supplementary Figure S9. Target plots (t-plots) for important miRNA targets confirmed by degradome sequencing. [file 44154_2024_158_MOESM9_ESM.png]

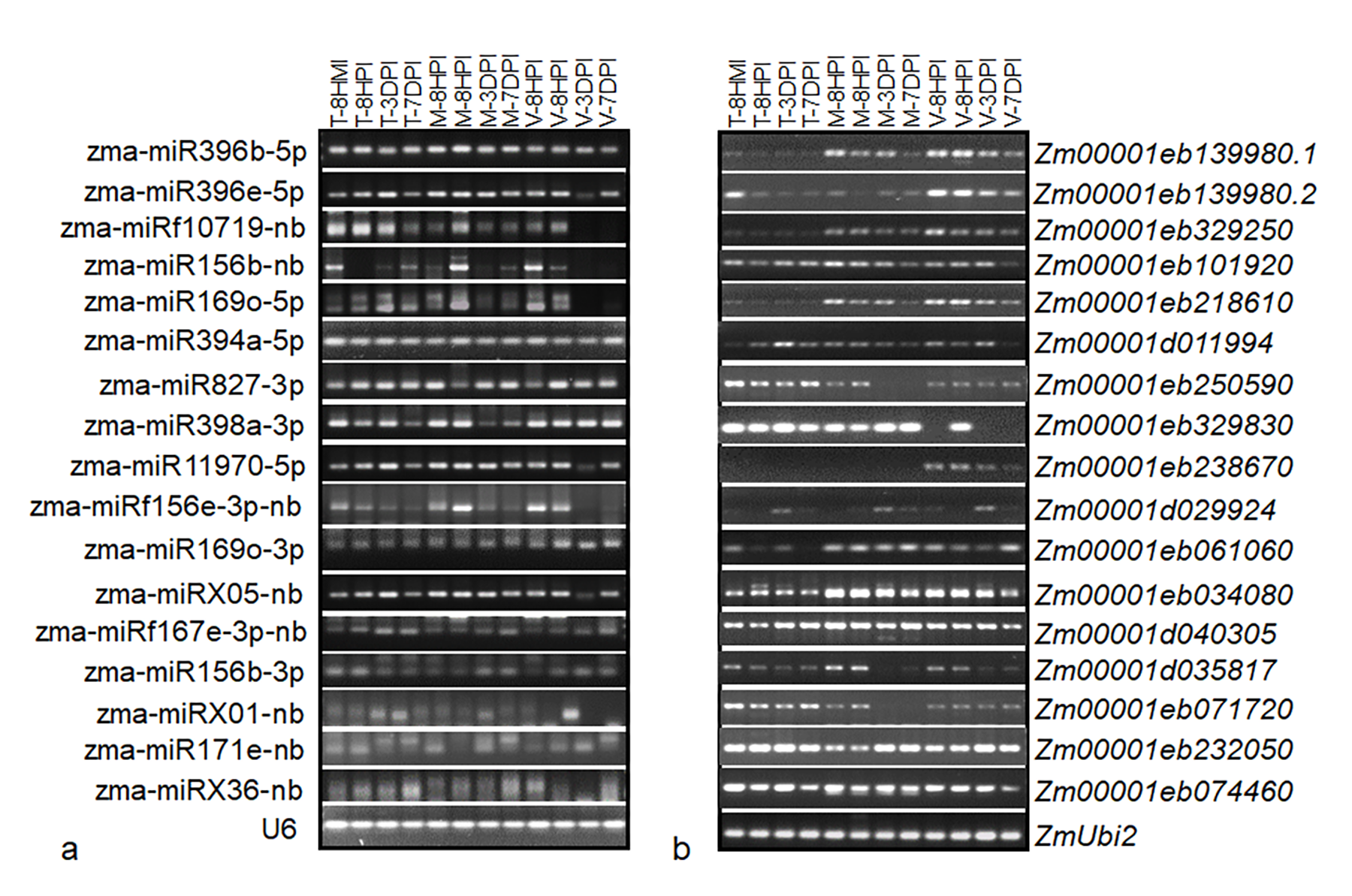

Supplement: Supplementary file 10 — Additional file 10:Supplementary Figure S10. Reverse transcription PCR showing expression pattern of miRNAs (a) and corresponding targets (b) in maize genotypes TZAR102, MI82, and Va35 at 8 h post mock inoculation, 8 h, 3 d, and 7d post inoculation with Aspergillus flavus. T = TZAR102, M = MI82, V = Va35, 8HMI = 8 h post mock-inoculation, 8 HPI = 8 h post inoculation with Aspergillus flavus, 3DPI = 3 d post inoculation with A. flavus, 7DPI = 7 d post inoculation with A. flavus [file 44154_2024_158_MOESM10_ESM.png]
